# Spatial transcriptomics reveals the mechanistic role of lactate metabolism in the pancreatic ductal adenocarcinoma microenvironment

**DOI:** 10.3389/fimmu.2026.1743187

**Published:** 2026-02-13

**Authors:** Fan Gao, Zhe Tang, Jie Lian, Luting Zhang

**Affiliations:** 1 Department of Hepatobiliary and Pancreatic Surgery, Shangyu People’s Hospital of Shaoxing, Shaoxing University, Shaoxing, Zhejiang, China; 2 Department of Pathology, Shangyu People’s Hospital of Shaoxing, Shaoxing University, Shaoxing, Zhejiang, China

**Keywords:** biomarkers, lactatemetabolism, pancreatic ductaladenocarcinoma, prognostic model, spatialtranscriptomics

## Abstract

**Background:**

Pancreatic ductal adenocarcinoma (PDAC), an aggressive cancer with poor prognosis, poses major challenges owing to late diagnosis and limited response to current therapies. However, the identification of candidate drugs through multi-omics analyses and therapeutic peptides targeting key molecular pathways may provide improved outcomes. Although lactate metabolism is a critical factor in tumor progression, affecting cell proliferation, metastasis, and immune evasion, its role in PDAC—particularly within the tumor microenvironment, remains underexplored.

**Objectives:**

This study investigated lactate metabolism in PDAC using high-throughput transcriptomic sequencing and single-cell transcriptomic analysis.

**Methods:**

Lactate metabolism–related gene expression was analyzed in tumor cells and their microenvironment, and correlations with patient prognosis were determined. Additionally, a machine learning–based prognostic model was established to identify lactate metabolism biomarkers for early diagnosis and personalized therapy.

**Results:**

Lactate metabolism significantly impacted the survival of patients with PDAC (n = 92; log-rank test, *p* < 0.05). Single-cell RNA and spatial transcriptomics analyses of 50, 795 cells from 8 PDAC samples revealed that 521 malignant cells exhibited hyperactive lactate metabolism (AUCell score comparison, *p* < 0.001). A prognostic model constructed from lactate metabolism–related genes using ensemble machine learning (StepCox + Enet, α = 0.5) effectively stratified patients into high- and low-risk groups across multiple cohorts (ICGC: n = 92; GSE28735: n = 45; GSE62452: n = 69; GSE183795: n = 139; all log-rank *p* < 0.05). Key prognostic genes identified included lysozyme (*LYZ*) and polymeric immunoglobulin receptor, which were significantly associated with patient survival (univariate Cox regression, *p* < 0.05). These genes may serve as clinical biomarkers of PDAC.

**Conclusions:**

This study provides insights into PDAC metabolic features and highlights lactate metabolism as a potential therapeutic target. The identified biomarkers could facilitate early diagnosis and improve treatment strategies, ultimately enhancing patient outcomes.

## 1 Introduction

Pancreatic ductal adenocarcinoma (PDAC) is one of the deadliest solid malignancies, with 5-year survival rates <10% despite multimodal therapies (1). It is marked by poor prognosis and a substantial global economic burden on healthcare systems (2). PDAC’s complexity arises from late diagnosis and resistance to conventional therapies, leading to limited treatment options and poor outcomes (3). Current diagnostic and therapeutic approaches, including surgery, chemotherapy, targeted therapy, and immunotherapy, remain only modestly effective, primarily due to tumor heterogeneity and a dense stromal microenvironment that restricts drug delivery (4–9). Moreover, existing treatments fail to address the metabolic reprogramming and cellular interactions driving PDAC progression, leaving major gaps in our understanding of the disease’s biology (10).

Studying lactate metabolism–related targets in PDAC is essential because they play a central role in tumor progression. PDAC cells undergo extensive metabolic reprogramming, frequently upregulating glycolysis to generate excess lactate even under normoxic conditions, a phenomenon known as the Warburg effect (11). Lactate, a key metabolic byproduct, shapes the tumor microenvironment (TME) by promoting acidosis, suppressing immune cell function, and driving angiogenesis and extracellular matrix remodeling, which together support tumor growth, invasion, and metastasis (12). Intensive research on the core enzymes and transporters involved in lactate metabolism, such as lactate dehydrogenase A/B (LDH A/B) and monocarboxylate transporters (MCTs), has identified promising therapeutic targets. Inhibiting these enzymes can disrupt cancer cell energy supply and metabolic balance, thereby slowing tumor progression (13, 14). Targeting lactate metabolism not only suppresses cancer cell proliferation but also enhances immunotherapy efficacy by reprogramming the TME. This strategy boosts immune cell antitumor activity, mitigates PDAC’s resistance to chemotherapy and radiotherapy, and improves therapeutic outcomes (15). Additionally, the expression levels of lactate and related enzymes function as diagnostic and prognostic biomarkers, aiding clinicians in assessing disease progression and personalizing treatment strategies. In summary, investigating lactate metabolism elucidates key metabolic mechanisms in PDAC, reveals multiple therapeutic targets, and holds promise for improving patient prognosis, extending survival, and reducing mortality through novel clinical interventions.

The integration of single-cell RNA sequencing (scRNA-seq) with spatial transcriptomics and machine learning has deepened our understanding of the TME (16, 17). This combined approach enables detailed characterization of cellular heterogeneity and complex intercellular interactions within the TME (18, 19). Furthermore, spatial transcriptomics maps gene expression within tissue architecture, clarifying how spatial organization influences cell-cell communication and tumor behavior (20). This study aimed to analyze lactate metabolism–associated cell subtypes in PDAC using single-cell and spatial transcriptomics, supported by evidence highlighting lactate’s importance in PDAC progression. By investigating the roles of these subtypes within the TME and their prognostic relevance, we sought to identify potential biomarkers and therapeutic targets. The integration of single-cell and spatial transcriptomics enhances our understanding of PDAC heterogeneity and underscores lactate metabolism’s pivotal role in tumor biology. Ultimately, this study provides novel insights into the mechanisms driving PDAC progression and identifies innovative strategies to improve patient outcomes (**Supplementary Figure 1**).

## 2 Materials and methods

### 2.1 Data acquisition and processing

Transcriptomic data were obtained from two publicly available databases: the International Cancer Genome Consortium (ICGC; https://dcc.icgc.org/) and the Gene Expression Omnibus (GEO; https://www.ncbi.nlm.nih.gov/geo/). Gene expression profiles and clinical data for PDAC were retrieved from ICGC, including 92 PDAC samples. Additional transcriptomic datasets were downloaded from GEO using the R package GEOquery. The included datasets were as follows: GSE28735 (45 PDAC tissue and 45 adjacent nontumor samples), GSE62452 (69 PDAC tissue and 61 adjacent nontumor samples), and GSE183795 (139 PDAC tissue, 102 adjacent nontumor samples, and 3 normal pancreatic ductal tissue samples). All datasets were processed according to the respective database access guidelines, ensuring compliance with ethical and data usage requirements. Through the Molecular Signatures Database (MSigDB) website (https://www.gsea-msigdb.org/gsea/msigdb/index.jsp), 320 lactate metabolism–related genes were identified (**Supplementary Table S1**).

### 2.2 Download and processing of single-cell sequencing data

The GEO database was used to retrieve scRNA-seq data related to lactate metabolism. Specifically, dataset GSE205354, comprising eight PDAC samples, was used. Raw scRNA-seq data from GSE205354 were processed using the Seurat package (v4.2.0) in R. Data preprocessing followed these criteria: genes with undetected expression were excluded; cells expressing >500 genes were retained; cells with <10, 000 unique molecular identifiers (UMIs) were removed; and cells with >25% mitochondrial gene expression were excluded. Data normalization was performed using the *NormalizeData* function in Seurat. After normalization, highly variable genes were identified by evaluating mean expression versus dispersion. Principal component analysis (PCA) was conducted, and significant principal components (PCs) were used for graph-based clustering.

To correct for batch effects, the Harmony method was applied for sample integration. Clustering was performed using the FindClusters function with the shared nearest neighbor modularity algorithm (resolution = 1.2) across the first 18 PCs. Clustering results were visualized using t-distributed stochastic neighbor embedding (t-SNE) via the RunTSNE function, and cell aggregation was visualized based on the t-SNE-1 and t-SNE-2 axes. Differentially expressed genes (DEGs) among clusters were identified using the FindAllMarkers function in Seurat with default settings. DEGs were selected based on expression in >25% of cells within a cluster and a log-fold change (logFC) >0.25, as recommended in Seurat documentation. The proportions of cell types were calculated using known cell type–specific biomarkers.

### 2.3 Download and processing of spatial transcriptomic sequencing data

Spatial transcriptomic data related to PDAC were obtained from GEO. The GSE203612 dataset, including 31 cancer-related spatial transcriptomic samples generated using various sequencing methods, was retrieved. This study focused on GSM6177614, obtained using the 10X Genomics Visium platform. Raw spatial transcriptomic data from GSM6177614 were processed using Seurat in R. Data were loaded using the Load10X_Spatial function, retaining spots with at least 500 detected genes. Normalization was conducted using SCTransform, followed by PCA performed via RunPCA. The optimal number of PCs was determined using the ElbowPlot function, and FindNeighbors was applied to compute pairwise cell neighborhood relationships.

Spatial expression patterns were visualized using SpatialPlot. Expressed UMIs and genes (nGenes) per spot were examined to assess sequencing quality, focusing on detected genes per UMI and UMIs per cell. Clustering was performed using the first 18 PCs at a resolution of 0.4, yielding 5 clusters. Clusters were annotated based on hematoxylin and eosin (HE) staining images and known cell type–specific biomarkers. DEGs among cell clusters were identified using the FindAllMarkers function based on normalized gene expression data.

### 2.4 AUCell-based screening of cells with active lactate metabolism

For single-cell analysis, each cell was scored using the AUCell R package (v1.24.0), which applies gene set enrichment analysis (GSEA). Gene expression ranking per cell was determined based on the area under the curve (AUC) values of 320 lactate metabolism–related genes (**Supplementary Table S1**). The AUC represents the proportion of highly expressed genes within a set, with higher AUC values indicating greater activity. The AUCell_exploreThresholds function was employed to establish thresholds for identifying lactate metabolism–active cells.

AUC scores were visualized using t-SNE, implemented via the *ggplot2* R package (v3.3.5), to represent activated cell clusters and preserve local data structures. This method maps high-dimensional data to two or three dimensions, enabling visualization of underlying patterns. For spatial transcriptome analysis, the SPATA2 R package (v0.1) was used to visualize spatial expression of lactate metabolism–associated gene sets. The plotSurface2 function in SPATA2 was employed with the parameters smooth = TRUE and smooth span = 0.2 to generate a smooth surface representation of the spatial gene expression.

### 2.5 InferCNV screening for malignant cells

The InferCNV R package (v1.10.1) was used to evaluate somatic large-scale chromosomal copy number variations (CNVs) in ductal cells based on single-cell gene expression data. This analysis aimed to identify potential malignant changes by comparing tumor and normal ductal cells through single-cell CNV profiling. Macrophages, endothelial cells, and stellate cells were selected as reference cell types. Iterative clustering analysis was conducted to distinguish malignant from nonmalignant cells. CNV scores were calculated to classify these populations by chromosomal alterations, an approach widely used in single-cell sequencing to differentiate tumor from normal cells and assess tumor heterogeneity.

### 2.6 Consensus clustering analysis

Consensus clustering, a robust unsupervised method, identifies stable and reproducible clusters in high-dimensional data through iterative similarity assessment. ConsensusClusterPlus (v1.58.0) was applied in R to classify PDAC samples based on lactate metabolism gene expression profiles. Samples were stratified into two clusters (Cluster 1 and Cluster 2) according to their consensus matrices and cumulative distribution function curves.

### 2.7 Building prognostic models using ensemble machine learning methods

To evaluate the prognostic value of lactate metabolism–related DEGs in PDAC, the R package *limma* was employed to identify DEGs between two groups: 41 samples with low lactate metabolism scores and 51 samples with high scores (|logFC| > 1.2, *p* <.05). Additional DEG identification was conducted via single-cell analysis, comparing malignant and normal cells as well as high-risk malignant lactate metabolism cells and their counterparts. Genes common to all three DEG sets were defined as bidirectional expression genes associated with lactate metabolism in PDAC. Univariate Cox regression analysis was then performed to assess associations between these DEGs and overall survival (OS) in each tumor cohort. Genes with *p* <.05 were considered significantly associated with OS and selected for further analysis, consistent with studies reporting improved PDAC survival for specific genetic profiles.

To construct a prognostic model based on lactate metabolism scores with high accuracy and stability, 10 machine learning algorithms were integrated: Random Survival Forest (RSF), ElasticNet (Enet), Lasso, Ridge, StepCox, CoxBoost, Cox partial least squares regression (plsRcox), supervised principal components (SuperPCs), generalized boosted regression modeling (GBM), and survival support vector machine (survival-SVM). Model performance was evaluated using cross-validation and by calculating accuracy, precision, recall, and F1 score. Lasso, StepCox, CoxBoost, and RSF incorporated built-in feature selection.

The RSF model was implemented using the randomForestSRC package in R. Enet, Lasso, and Ridge were executed using glmnet, with the L1-L2 balance parameter (α) varied from 0 to 1 at 0.1 intervals. StepCox was performed using the survival package in R with the stepwise search modes “both, “ “backward, “ and “forward.” CoxBoost was implemented using the CoxBoost package (v1.4.0) in R, with the optimal penalty and optimal step number determined via the optimCoxBoostPenalty and cv.CoxBoost functions, respectively. To execute plsRcox, the plsRcox package (v1.8.0) was implemented in R, using cv.plsRcox to determine the number of extracted components. SuperPC was implemented through the superpc package in R, with the superpc.cv function selecting the best threshold. GBM was executed using the gbm package in R, with the cv.gbm function employed to select the model with the lowest cross-validation error. Finally, survival-SVM was implemented via the survivalsvm package in R.

Model training was conducted on the ICGC-PDAC cohort and validated on independent datasets (GSE28735, GSE62452, and GSE183795). The model exhibiting the highest average concordance index (C-index), i.e., StepCox [backward] + Enet [α = 0.5], was chosen as the final model, as it demonstrated superior predictive accuracy. Kaplan–Meier analysis was used to evaluate this model’s prognostic value. Immunohistochemical staining images of the gene protein products in head and neck squamous cell carcinoma samples were obtained from the Human Protein Atlas (HPA; http://www.proteinatlas.org).

### 2.8 Spatial transcriptome analysis by deconvolution

Spatial transcriptomics datasets generated by 10X Genomics VISIUM technology initially lacked single-cell resolution, a limitation addressed via Visium HD. In spatial transcriptomics, gene expression at each spatial spot represents multiple cells and can be analyzed similarly to small RNA-seq datasets. To estimate cell type proportions at each spatial location, deconvolution methods are necessary. The spatial coordinates of individual cells were obtained through joint analysis of scRNA-seq data and spatial transcriptomic data using the CellTrek package in R[35, 314, 812] with default parameters. The run_kdist function in CellTrek was employed to calculate spatial k-distances between cell types, and cell colocalization patterns were examined using the SColoc function within CellTrek.

### 2.9 Cell communication analysis with ligand–receptor expression

Cell communication analysis was performed to identify signaling pathways by quantifying ligand–receptor pair expression across cell types. The CellChat package (v1.1.3) was employed to detect afferent and efferent signaling patterns, measure communication probabilities, and calculate information flow for each pathway. In PDAC samples, CellChat analysis focused on how lactate metabolism modulates intercellular interactions within the TME. The strength of each signaling pathway was examined using multidimensional clinical proteomics, and key pathways were selected for visualization. Default CellChat parameters were applied with significance set at *p* <.05, and multiple testing corrections were performed using the Benjamini–Hochberg method.

### 2.10 Single-cell regulatory network inference and clustering analysis

Single-cell regulatory network inference and clustering (SCENIC) analysis was performed using the pySCENIC Python package (v0.12.0). Coexpression modules were first identified between transcription factors (TFs) and candidate target genes. The RcisTarget function was then used to detect enriched motifs in each module, defining TFs and their direct target genes. Subsequently, the AUCell function was employed to evaluate module activity in individual cells, and module cell type–specificity was determined using the module specificity score.

### 2.11 Gene ontology and kyoto encyclopedia of genes and genomes pathway enrichment analysis

DEGs were identified between active and inactive lactate cells using the FindAllMarkers function with a threshold of |log2Fold Change| > 0.25 and *p* <.05. DEGs between PDAC and control samples were determined using the limma package with a screening threshold of |log2Fold Change| > 2 and *p* <.05. Two DEGs were further analyzed in PDAC. Gene Ontology (GO) enrichment analysis was used to assess biological process (BP), molecular function (MF), and cellular component (CC) enrichment. Kyoto Encyclopedia of Genes and Genomes (KEGG) pathway analysis was employed to identify significantly enriched metabolic pathways in the DEG list. Both GO and KEGG analyses were conducted via the clusterProfiler package (v4.2.2) in R, using a significance threshold of *p* <.05.

### 2.12 GSEA

GSEA was conducted to determine whether predefined gene sets displayed significant expression differences between biological states. The limma package was used to analyze the differential expression of prognosis-related genes and calculate log2FC. GSEA was performed in clusterProfiler using a ranked gene list based on log2FC values. Each analysis included 1, 000 gene set permutations. The c2.cp.kegg.v7.5.1.symbols gene set from MSigDB served as the reference. Gene sets with *p* <.05 were considered significantly enriched.

### 2.13 Drug sensitivity analysis

Drug sensitivity data were obtained from the Genomics of Drug Sensitivity in Cancer database (https://www.cancerrxgene.org/), including half-maximal inhibitory concentration (IC50) values and corresponding gene expression profiles. IC50 values provide a quantitative measure of drug efficacy, representing the concentration required to inhibit 50% of cancer cell viability. To predict therapeutic sensitivity in PDAC patients, the R package oncoPredict (v0.2) was employed. This tool integrates gene expression and drug response data to estimate PDAC sample sensitivity to chemotherapeutic agents.

### 2.14 Immune checkpoint analysis

Immune checkpoints are regulatory molecules expressed on immune cells that modulate immune activation levels and prevent excessive immune responses. The expression profiles of immune checkpoint genes and their associations with prognosis-related genes were examined in clusters 1 and 2. The Wilcoxon rank-sum test, a nonparametric method for comparing medians between independent samples, was performed via the *wilcox.test* function in R’s *stats* package.

### 2.15 Tumor immune dysfunction and exclusion analysis

To predict patient response to immunotherapy, the tumor immune dysfunction and exclusion (TIDE) algorithm (http://tide.dfci.harvard.edu/) was employed. Correlations among immune checkpoint genes, key genes, and TIDE scores were evaluated using Pearson correlation analysis, following the TIDE framework for assessing immune checkpoint blockade efficacy. Correlation coefficients and their statistical significance were computed using functions from the R package Hmisc.

### 2.16 Pan-cancer analysis

Pan-cancer analysis was performed to evaluate the expression, prognostic significance, and immune associations of key genes across 33 cancer types. This approach has previously been used to analyze tumor coagulome genes and their link to the TME, as well as 33 human cancers to assess the immunotherapeutic value of specific genes. Genome-wide expression and clinical data were obtained from The Cancer Genome Atlas using the R package *TCGAbiolinks* (v2.25.0). The expression differences of key genes across the 33 cancer types were analyzed. For survival analysis, a univariate Cox regression model was employed to assess the relationship between key gene expression and tumor prognosis. Immune infiltration scores for each tumor sample were calculated using the ssGSEA algorithm, and correlations between immune infiltration scores and key gene expression levels were determined.

### 2.17 Cell culture

The PDAC-representative cell lines ASPC-1, MIA-PACA2, and PANC-1 and the controls human pancreatic ductal epithelium (HPDE) and SW1990 were obtained from Pricella (Wuhan, China). Cells were cultured in RPMI-1640 medium supplemented with 10% fetal bovine serum at 37°C in a humidified incubator with 5% CO_2_.

### 2.18 Reverse transcription polymerase chain reaction

Reverse transcription polymerase chain reaction (RT-PCR) was conducted in four stages: cell seeding, RNA extraction, reverse transcription, and quantitative RT-PCR (qRT-PCR). PDAC cell lines (ASPC-1, HPDE, MIA-PACA2, PANC-1, and SW1990) were seeded in 6-well plates and cultured in drug-containing serum. RNA was extracted following standard protocols involving cell washing, lysis, centrifugation, and RNA precipitation. For reverse transcription, the HiScript III RT SuperMix Kit was used to eliminate genomic DNA. Subsequently, qRT-PCR was performed using ChamQ Universal SYBR qPCR Master Mix, with glyceraldehyde-3-phosphate dehydrogenase (GAPDH) as an internal reference. Relative expression levels were calculated using the 2^-△△Ct^ method.

### 2.19 Western blot

Western blotting was performed through sequential steps: cell culture, protein extraction, gel preparation, electrophoresis, protein transfer, blocking, antibody incubation, and imaging. Total proteins from ASPC-1, HPDE, MIA-PACA2, PANC-1, and SW1990 cells were extracted using radioimmunoprecipitation assay (RIPA) lysis buffer. Protein lysates were separated via sodium dodecyl sulfate–polyacrylamide gel electrophoresis, transferred onto polyvinylidene fluoride membranes, blocked, and incubated sequentially with primary and secondary antibodies. Protein bands were visualized using a fluorescence scanner.

### 2.20 Clinical sample collection

Paraffin-embedded tissue samples from patients with PDAC were collected from Shangyu People’s Hospital of Shaoxing, Shaoxing University, during June 2020–December 2024.

### 2.21 Immunohistochemical analysis

Immunohistochemistry was performed through standard steps of deparaffinization, hydration, antigen retrieval, and antibody incubation. Paraffin sections were deparaffinized in xylene and rehydrated through graded ethanol solutions. Antigen retrieval was performed using ethylenediaminetetraacetic acid (EDTA) or citrate buffer, with tissue sections heated in a microwave, maintained at boiling, and then cooled at room temperature. Endogenous peroxidase activity was blocked, followed by serum blocking. Sections were incubated with primary and secondary antibodies, and staining was visualized using 3, 3’-diaminobenzidine (DAB). Staining intensity, indicative of antigen abundance, was monitored microscopically. Sections were counterstained, dehydrated, mounted, and imaged for analysis.

The expression levels of PIGR protein were independently evaluated in a semi-quantitative manner by two senior pathologists using a double-blinded approach (unaware of the sample grouping information). The assessment integrated staining intensity and the percentage of positive tumor cells, with results classified into four grades: – (negative): no staining or positive cells < 5%; + (weak positive): pale yellow staining and positive cells ≥ 5%; ++ (moderate positive): brownish-yellow staining and positive cells ≥ 25%; +++ (strong positive): brown staining and positive cells ≥ 50%. The results from the two evaluators showed a high degree of concordance. For the few discrepant cases, a consensus was reached through joint re-evaluation and discussion, and the agreed-upon result was used as the final determination for subsequent statistical analysis.

### 2.22 Statistical analysis

All analyses were performed using R (v4.1.2). Survival differences between groups were assessed using the Kaplan–Meier method, with significance determined via the log-rank test. Survival curves were generated using the *survminer* package. Univariate and multivariate Cox regression analyses identified prognostic variables. Lasso regression was employed to select high-impact predictors of survival outcomes. Data visualization was performed using *ggplot2*, with risk and OS scores calculated using the *survival* package. Heatmaps were generated using *pheatmap*. Continuous variables were compared using two-tailed t-tests or one-way analysis of variance (ANOVA) for normally distributed data, with the Wilcoxon rank-sum test or Kruskal–Wallis test employed for non-normal distributions. *p* < 0.05 was considered statistically significant. All basic experiments were performed in triplicate, and results are presented as means ± standard deviations. Statistical analysis for these experiments was performed using GraphPad Prism 8. One-way ANOVA followed by Tukey’s *post hoc* test was used to compare group means, with *p* <.05 denoting statistical significance.

## 3 Results

### 3.1 Single-cell dimensionality reduction, clustering, and annotation

**Figure 1:**
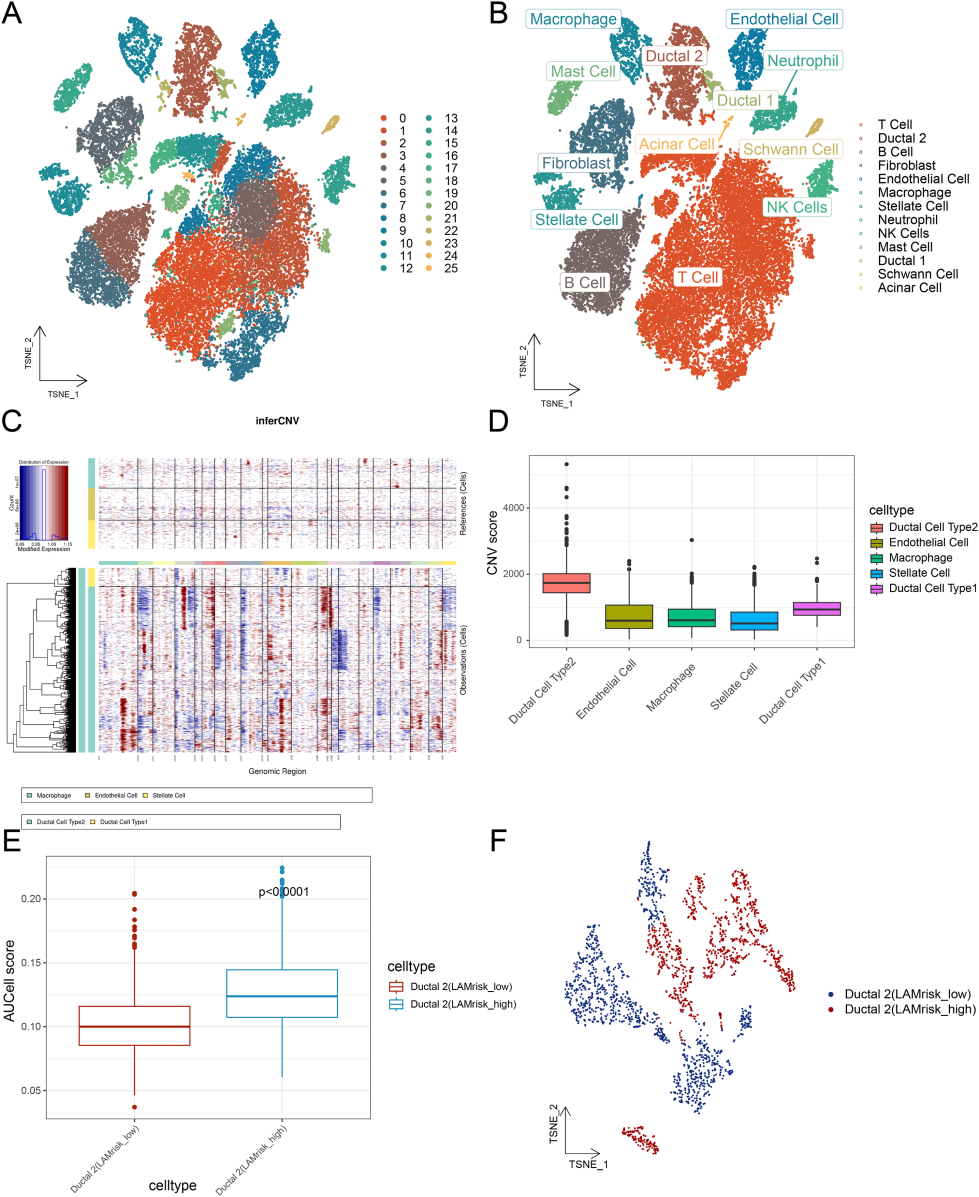
Single-cell dimensional reduction clustering of PDAC and identification of malignant cells based on lactate metabolism. **(A, B)** t-SNE plots showing the cluster distribution of PDAC cells. **(C)** InferCNV heat map illustrating the cell CNV scores. **(D)** Bar graphs showing CNV score differences among cell populations. **(E)** Histogram depicting differences in AUCell scores between lactate metabolism–active cell types. **(F)** t-SNE plot displaying the annotation results of lactate metabolism–active cells. PDAC, pancreatic ductal adenocarcinoma; t-SNE, t-distributed stochastic neighbor embedding; CNV, copy number variation.

We analyzed PDAC cell populations using the scRNA-seq dataset GSE205354. After quality control and doublet removal, 50, 795 cells were retained for downstream analysis. The dataset comprised eight samples, with a uniform distribution across these samples, indicating minimal batch effects and ensuring reliability for subsequent analysis. Clustering divided cells into 26 distinct clusters (**Figure 1A**), which were annotated based on gene expression profiles and cell-specific biomarkers (**Supplementary Table S2**). As shown in **Figure 1B**, we identified 13 major cell types: T cells, B cells, fibroblasts, endothelial cells, macrophages, stellate cells, neutrophils, natural killer (NK) cells, mast cells, ductal cells 1, ductal cells 2, Schwann cells, and acinar cells. Marker genes for each cell type were visualized using tools from scPlantDB (**Supplementary Figure 2A**). The proportional distribution of these cell types across tumor and normal samples is shown in **Supplementary Figures 2B, C**.

### 3.2 InferCNV identification of malignant and lactate-metabolic malignant cells

As PDAC originates from ductal cells, we applied inferCNV analysis to single-cell gene expression data to distinguish malignant from nonmalignant cells, employing macrophages, endothelial cells, and stellate cells as references. Unsupervised clustering based on CNV profiles identified ductal 2 cells as malignant owing to their elevated CNV levels (**Figures 1C, D**).

To identify malignant subsets with active lactate metabolism, we assessed gene expression patterns at the single-cell level. Based on the AUCell-determined threshold of lactate metabolic activity, ductal 2 cells were further classified, with cells exceeding this threshold categorized as active cells, showing 521 malignant cells with active lactate metabolism. The AUC scores of these cells were significantly higher than those of inactive cells (p <.001; **Figure 1E**). Dimensionality reduction using t-SNE further distinguished malignant cells by lactate-metabolic activity (**Figure 1F**).

Comparison of active malignant and other cells yielded 382 DEGs with significant differences (corrected *p* <.05, |log2FC| > 1; **Supplementary Table S3**). GO (**Supplementary Table S4**) analyses revealed DEG enrichment in response to steroid hormones, positive regulation of cytokine production, and adenosine triphosphate (ATP) metabolic processes (all BP), along with localization to cytosolic ribosomes and secretory granule lumen (both CC), and cadherin binding and major histocompatibility complex class II protein complex binding (both MF) (**Supplementary Figure 2D**). KEGG pathway analysis (**Supplementary Table S5**) identified enrichment in prion diseases, oxidative phosphorylation, and pancreatic secretion (**Supplementary Figure 2E**).

### 3.3 Consistent clustering identified lactate metabolism subgroups

**Figure 2:**
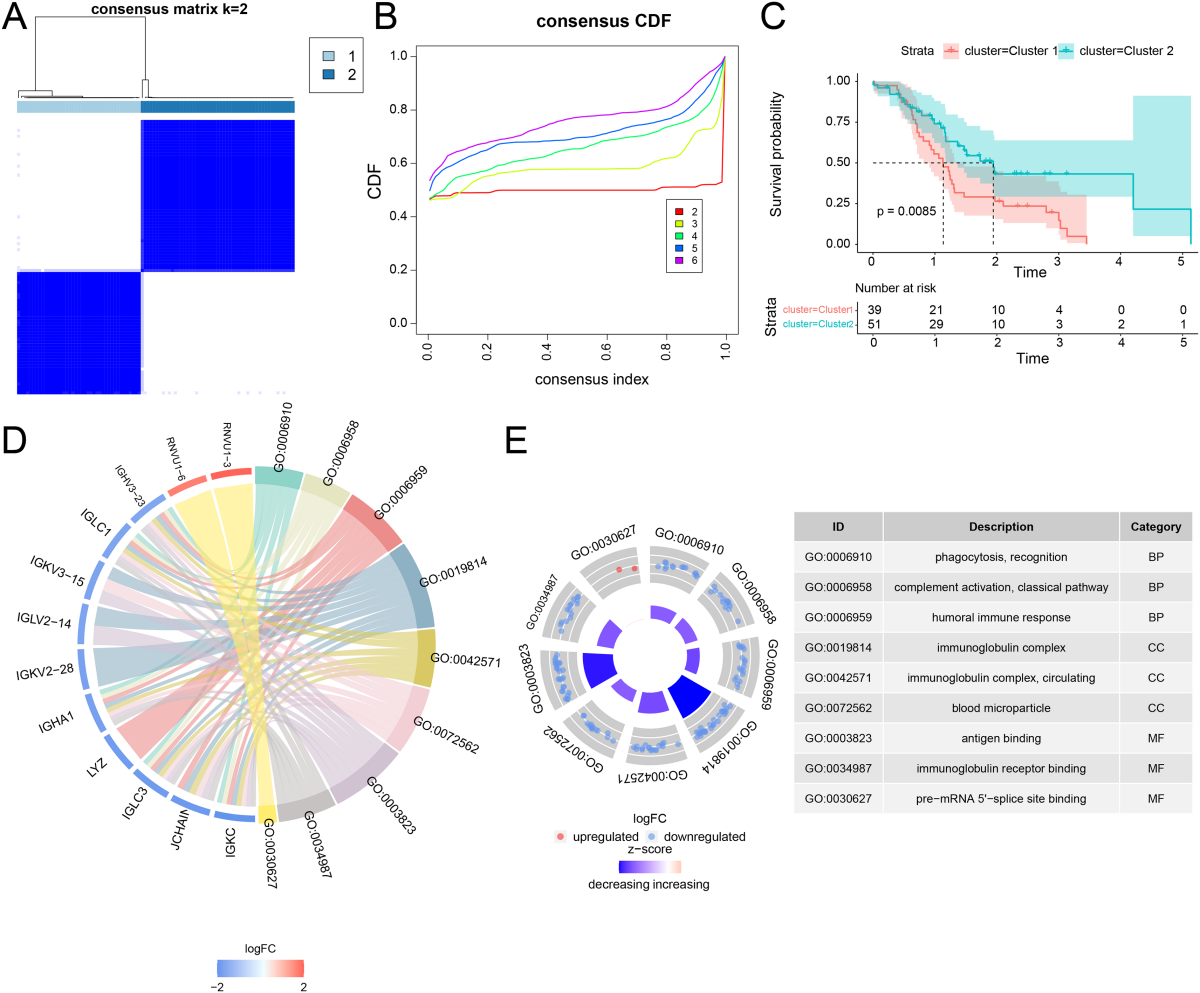
Differential analysis of lactate metabolism subtypes in patients with PDAC and corresponding changes in cell abundance. **(A)** Consensus clustering at *k* = 2. **(B)** CDF plot showing the consistency of cluster distribution in patients with PDAC. **(C)** Kaplan–Meier survival analysis comparing the estimated survival probabilities of different lactate metabolism subtypes. **(D)** Chord diagram showing GO enrichment of DEGs among subtypes. **(E)** Circular plot displaying significantly enriched GO pathways, along with a corresponding table of pathway numbers and pathway names. CDF, cumulative distribution function; PDAC, pancreatic ductal adenocarcinoma.

Unsupervised clustering of 320 key lactate metabolism–related genes defined two distinct PDAC subtypes (**Figure 2A**). The optimal clustering solution (k = 2) produced two stable and well-separated subgroups representing active lactate metabolism profiles (**Figure 2B**). Kaplan–Meier survival analysis revealed significant prognostic differences between the subtypes: patients in Cluster 2 demonstrated significantly improved survival rates compared with those in Cluster 1 (**Figure 2C**). Similarly, in a multiple myeloma study, Cluster 1 showed significantly lower lactate metabolism scores compared with Cluster 2 (**Supplementary Figure 3A**).

Differential expression analysis identified 69 DEGs between the two subgroups (corrected *p* <.05, |log2FC| > 1.2), with 26 upregulated and 43 downregulated in Cluster 1 (**Supplementary Table S6**). olcano plots and heatmaps illustrated these differences (**Supplementary Figures 3B, C**): the top five upregulated genes (RNVU 1-6, RNVU 1-1-3, RNVU 1-2, SNORA27, and RN7SL451P) and top five downregulated genes (JCHAIN, SLC4A4, LYZ, SLC40A1, and CLDN2) are shown in these plots (**Supplementary Figure 3B**). GO enrichment analysis (**Supplementary Table S7**) indicated that DEGs were enriched in phagocytosis, recognition, complement activation (classical pathway), and humoral immune response (all BP). In terms of cellular localization, enriched components included the immunoglobulin complex, circulating immunoglobulin complex, and blood microparticles (all CC). Enriched functions included antigen binding, immunoglobulin receptor binding, and peptidoglycan binding (all MF) (**Supplementary Figures 3D, E**).

### 3.4 Construction and validation of a prognostic model for lactate metabolism–related genes

**Figure 3:**
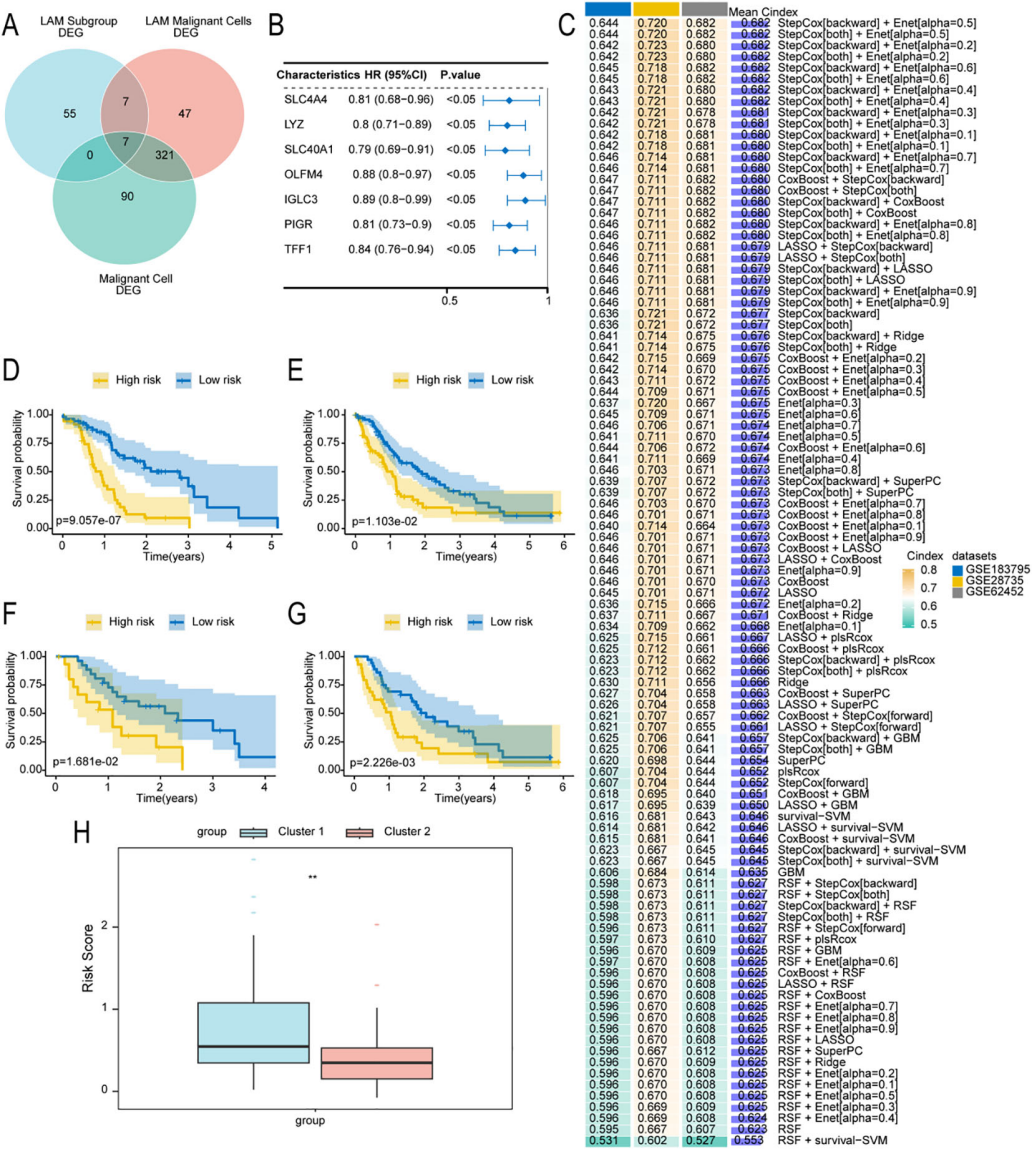
Construction of a prognostic model using machine learning. **(A)** Venn diagram showing the final key genes identified from multiple differential gene analyses. **(B)** Univariate Cox regression showing the association between key genes and prognosis. **(C)** Heatmap of the concordance index (C-index) values of 101 combined machine-learning algorithms across datasets, with the adjacent bar graph displaying the mean C-index values in the validation cohort. **(D–G)** Kaplan–Meier survival curves showing the relationship between risk score and overall survival in the International Cancer Genome Consortium cohort **(D)**, GSE28735 **(E)**, GSE62452 **(F)**, and GSE183795 **(G)** datasets. **(H)** Boxplot illustrating differences in risk scores among PDAC lactate-metabolism subtypes. PDAC, pancreatic ductal adenocarcinoma.

To identify key genes associated with lactate metabolism, we conducted screening by integrating single−cell data and transcriptomic data. The key genes were obtained from the intersection of three gene sets. The first set was derived from the comparison between PDAC and control groups in the transcriptomic data, representing the major genes identified at the transcriptome level that are associated with PDAC development (LAM subgroup DEGs, n=69). The second set came from differential analysis between ductal2 and all other cell types in the single−cell data, as ductal2 is considered malignant; this set represents the main genes related to tumorigenesis in ductal cells (LAM Malignant Cells DEGs, n=382). The third set of genes was obtained from the comparison between LAMrisk_high cells and all other cells; this set is defined as closely associated with the activation of lactate metabolism (Malignant Cell DEGs, n=418). Ultimately, seven overlapping genes were identified (**Figure 3B**).

A prognostic model was built using 10 machine learning algorithms applied to the ICGC-PDAC cohort and validated across three external datasets (GSE28735, GSE62452, and GSE183795). The model with the highest mean C-index across three validation cohorts was selected as optimal, as the C-index (ranging from 0 to 1) quantifies concordance between predicted and actual outcomes. The final StepCox [backward] + Enet [α = 0.5] model identified two signature genes, namely lysozyme (LYZ) and polymeric immunoglobulin receptor (PIGR), which were used to construct the prognostic model (**Figure 3C**; **Supplementary Table S9**). Survival analyses of the ICGC-PDAC and GSE28735, GSE62452, and GSE183795 validation datasets showed that higher risk scores were significantly associated with shorter survival times (**Figures 3D–G**). Moreover, risk scores differed significantly between Cluster 1 and Cluster 2 (*p* = .01; **Figure 3H**).

### 3.5 Screening of prognostic-related genes as lactate metabolism biomarkers

The expression levels of prognostic genes across lactate metabolism subtypes are shown in **Supplementary Figure 4A**. All prognostic genes displayed significant differential expression among the lactate metabolism subtypes. We further examined the correlations between prognostic genes and corresponding cell types, as shown in scatter plots (**Supplementary Figure 4B**). LYZ exhibited a significant positive correlation with B cells (R = 0.700, *p* <.001), ductal malignant hyperlactate metabolism cells (ductal 2, LAMrisk_high; R = 0.700, *p* <.001), and macrophages (R = 0.642, *p* <.001). Similarly, PIGR showed significant positive correlations with ductal 2 cells (LAMrisk_high: R = 0.717, *p* <.001; LAMrisk_low: R = 0.656, *p* <.001) and fibroblasts (R = 0.615, *p* <.001) (**Supplementary Figure 4B**). These findings highlight LYZ and PIGR as potential prognostic biomarkers for PDAC.

To further elucidate their mechanisms in PDAC, we performed survival analysis and single-gene GSEA on PDAC-associated tumor markers (**Supplementary Figures 4C–E**). Survival analysis revealed that higher LYZ and PIGR expression levels were associated with improved survival outcomes (**Supplementary Figures 4C, E**). GSEA showed that LYZ-associated genes were primarily enriched in the hallmark kirsten rat sarcoma viral oncogene homolog (KRAS) signaling up, hallmark IL2 STAT5 signaling, and hallmark adipogenesis pathways (**Supplementary Figure 4D**). In contrast, PIGR-associated genes were mainly enriched in the epithelial–mesenchymal transition, KRAS signaling up, and interferon gamma response pathways (**Supplementary Figure 4F**).

LYZ and PIGR protein expression levels were further validated using immunohistochemistry staining images obtained from the HPA database, confirming high-expression levels of both proteins in PDAC tissues at the translational level (**Supplementary Figures 4G, H**). Additional single-gene analysis results are provided in **Supplementary Table S10**.

### 3.6 Spatial transcriptomic heterogeneity analysis in PDAC

**Figure 4:**
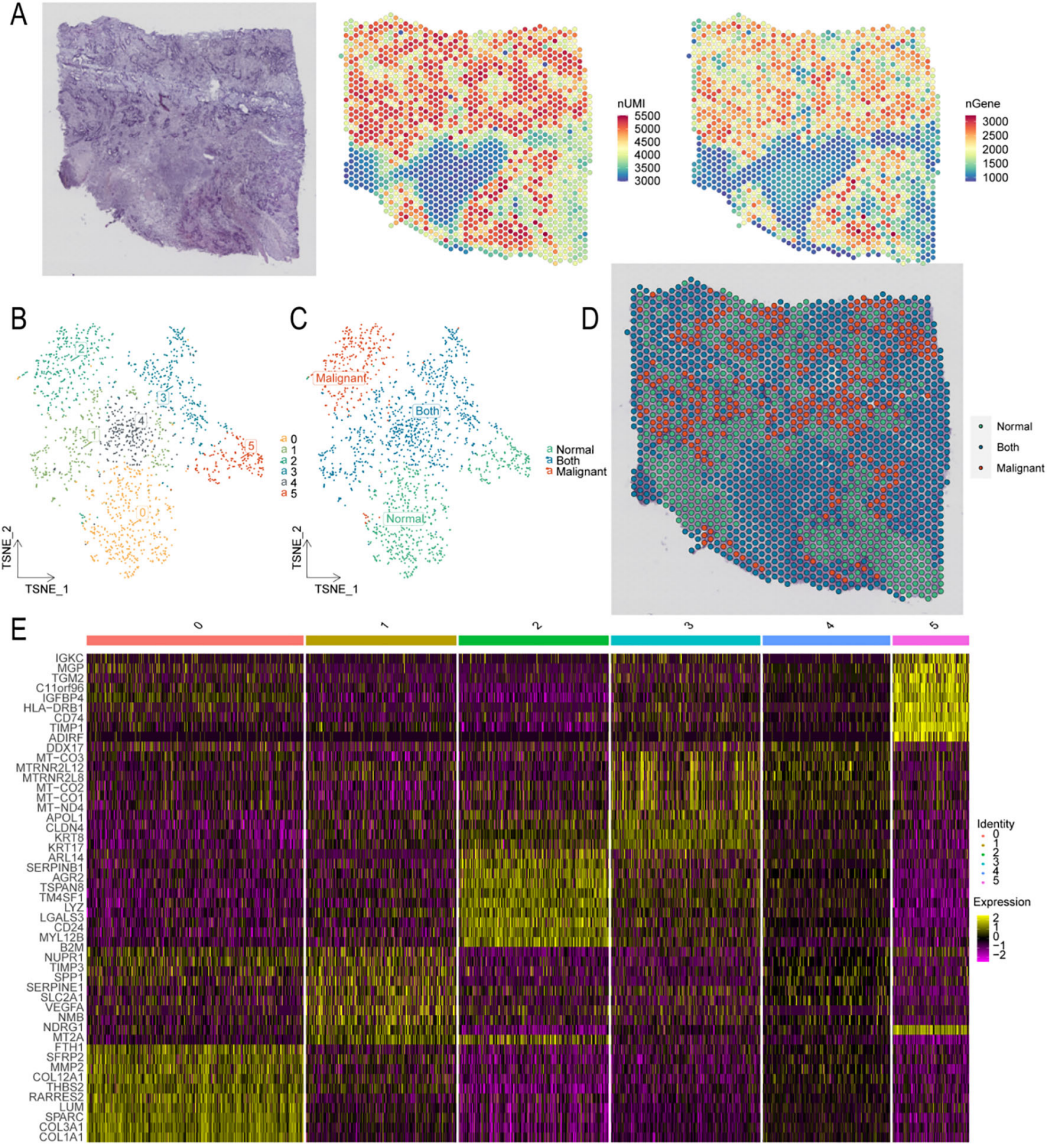
Spatial transcriptomic quality control and annotation. **(A)** HE-stained image and spatial maps showing the distribution of UMIs and gene spatial density. **(B)** t-SNE cluster plot. **(C)** Annotation of t-SNE dimensional reduction clustering. **(D)** Spatial distribution maps of different cell types. **(E)** Heatmap showing the 10 most significantly differentially expressed genes among clusters. HE, hematoxylin and eosin; UMI, unique molecular identifier; t-SNE, t-distributed stochastic neighbor embedding.

We next mapped the spatial distribution of pancreatic cells within the originating tissue. In the spatial transcriptomics sequencing (ST-seq) data, mitochondrial RNA accounted for <20% of total reads, indicating high data quality. More than 3, 000 genes were detected via ST-seq (**Figures 4A–C**). Clustering analysis grouped cells into five clusters (**Figure 4B**). Each cluster was annotated using gene expression features, cell-specific biomarkers, and HE staining. In differential analysis (**Figure 4E**), Cluster 2 predominantly expressed B2M, CD24, LGALS3, and TM4SF1, genes overexpressed in tumor and epithelial cells, particularly in ovarian cancer, and linked to tumor progression and metastasis. Thus, Cluster 2 was annotated as cancer cells of epithelial origin. Clusters 0 and 5 mainly expressed TGM2, IGFBP4, and HLA-DRB1 as well as fibrous markers, such as CD74, characteristic of epithelial cells and adipocytes, and were annotated as normal cells. Clusters 1, 3, and 4 were primarily enriched in the genes NUPRI, TPM3, SERPINE1, and SLC2A1, biomarkers of pre-existing tumor and normal cells, and were classified as intermixed cells. Ultimately, the dataset was categorized into three groups: tumor, normal, and intermixed cells (**Figures 4C, D**).

**Figure 5:**
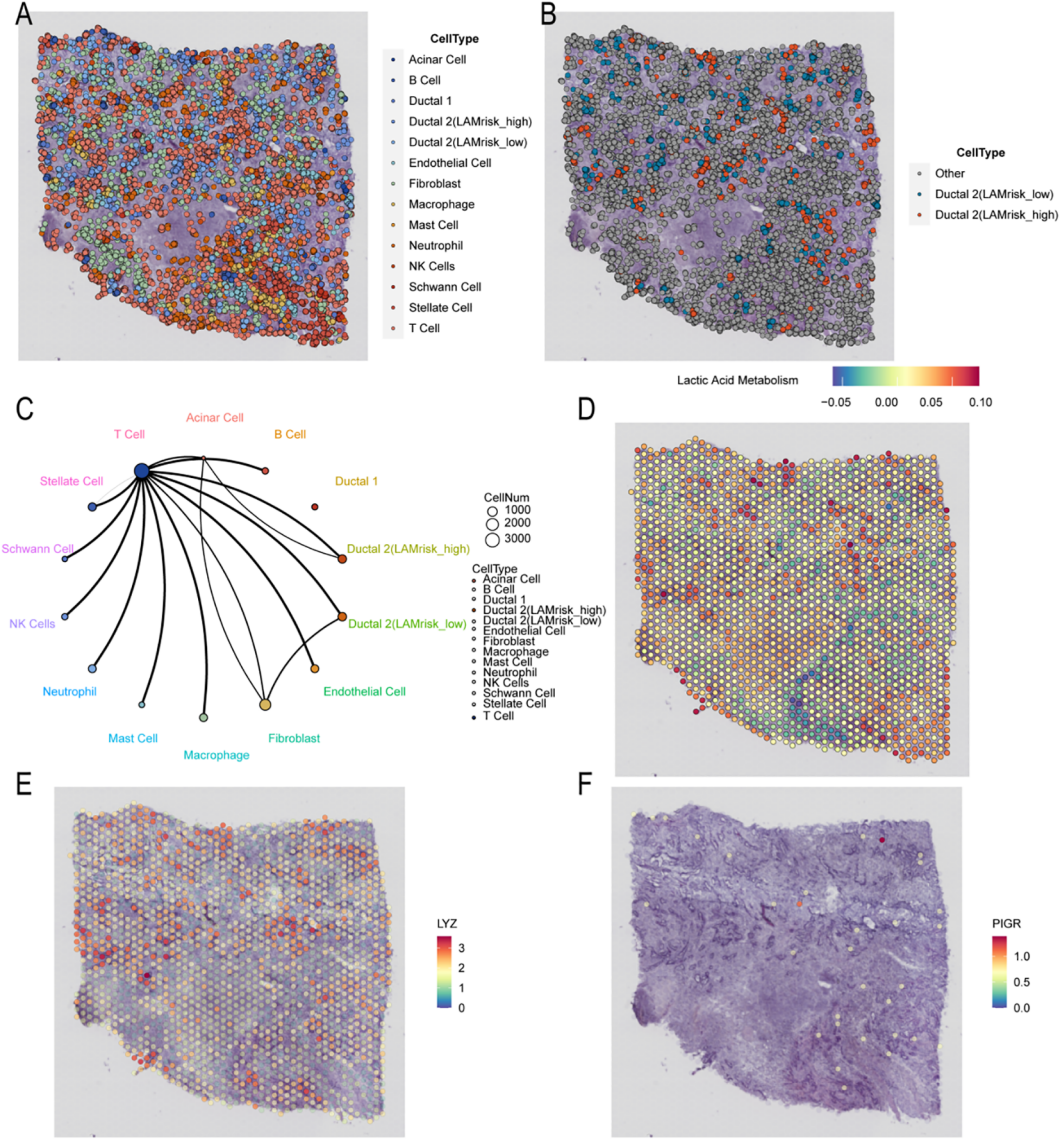
Integrated analysis of single-cell and spatial transcriptomic data. **(A)** Spatial transcriptome map showing the localization of different cell types. **(B)** Spatial mapping comparing malignant bile duct hyperlactate cells and malignant cells in the spatial transcriptome. **(C)** Network plots illustrating colocalization relationships among different cell types in spatial context. **(D)** Spatial variation of the lactate metabolism score. **(E)** Spatial mapping of *LYZ* gene expression. **(F)** Spatial mapping of *PIGR* gene expression. LYZ, lysozyme; PIGR, polymeric immunoglobulin receptor.

Using CellTrek analysis, we inferred the single-cell composition of each spatial spot to map all scRNA-seq clusters. The identified cell types, i.e., fibroblasts, endothelial cells, stellate cells, NK cells, mast cells, donor cells, acinar cells, and bile duct cells with hyperlactate metabolism, served as key indicators of malignancy. Notably, cells with hypolactate metabolism reflected the tumor’s metabolic and oxygenation state. Immune cells, including macrophages, neutrophils, NK cells, T lymphocytes, and B lymphocytes, were primarily distributed in intermixed clusters (**Figure 5A**). The colocalization patterns of different cell types are shown in **Figures 5B, C**, revealing that malignant bile duct cells with elevated lactate metabolism primarily colocalized with acinar and T cells.

We calculated the lactate metabolism score for each cell type using the AddModuleScore function with 320 characteristic lactate metabolism–related genes, enabling estimation of lactate metabolism levels across tissues. As shown in **Figure 5D**, cells with high lactate metabolism were localized in regions corresponding to malignant bile duct cells with elevated metabolic activity. Furthermore, we investigated the expression patterns of prognostic genes across various tissue types and found that LYZ expression was predominantly enriched in tumor tissues (**Figures 5E, F**).

### 3.7 Cell communication analysis

**Figure 6:**
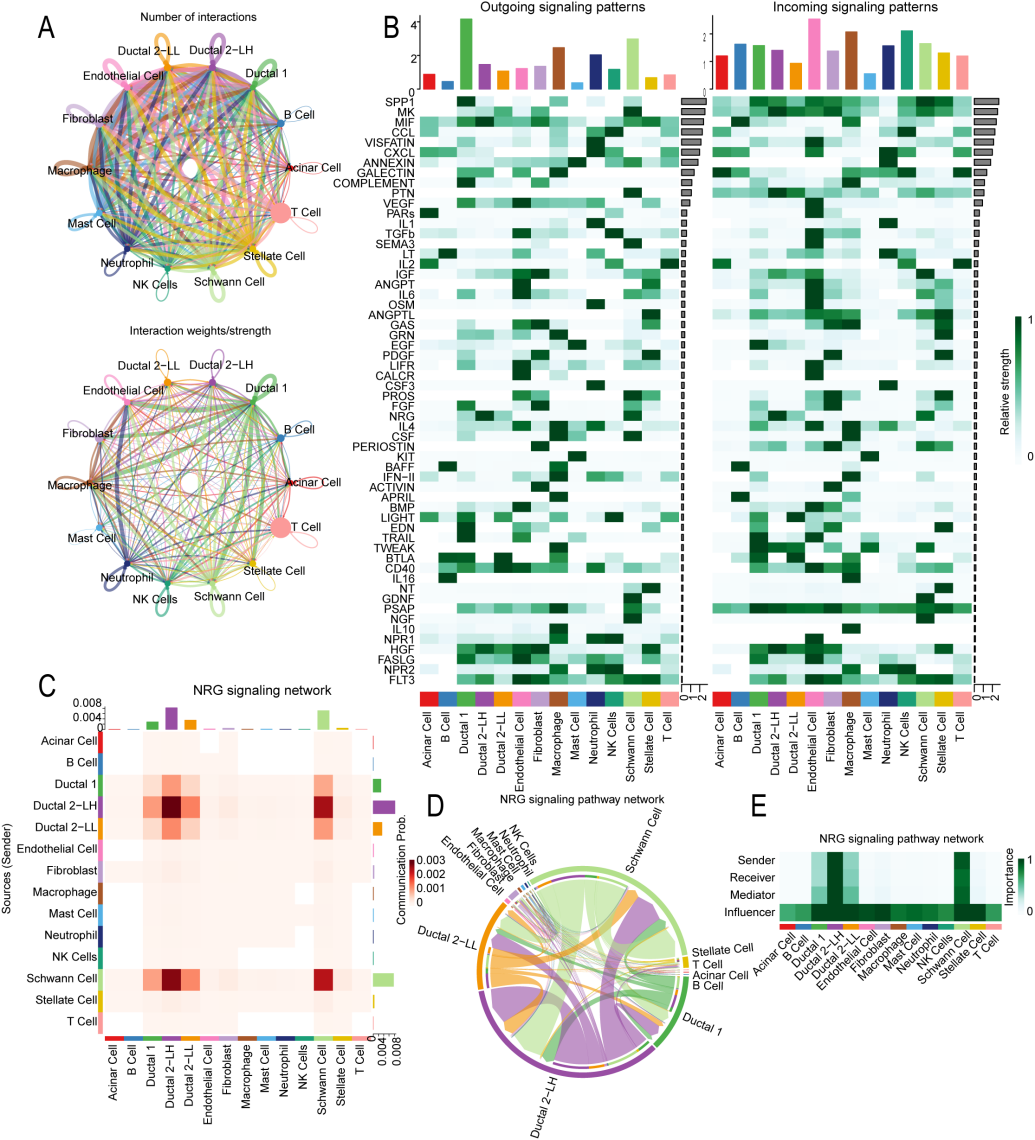
Results of cell-to-cell communication analysis. **(A)** Network plots showing the number and strength of interactions among cell types. **(B)** Heatmap visualizations of potential afferent and efferent signaling pathways between cells. Ductal 2-LH refers to malignant bile duct cells with high lactate metabolism, and Ductal 2-LL refers to those with low lactate metabolism. **(C)** Heatmap showing cell population interactions mediated by the NRG signaling pathway. **(D)** Chord diagram illustrating cell population interactions mediated by NRG signaling across different cell types. **(E)** Heatmap showing the roles of individual cell populations within the signaling pathway.

We applied the CellChat algorithm to analyze intercellular signaling networks based on single-cell transcriptomic data, aiming to predict pathological changes in cell-to-cell communication in PDAC. This analysis revealed that malignant cells with low lactate metabolism exhibited an increase in communication events and higher interaction intensities compared with other cell types (**Figure 6A**).

To further quantify intercellular communication, we calculated the information flow for each signaling pathway across cell-type pairs, enabling assessment of communication probabilities and differences in information flow between cells with high and low lactate metabolism levels (**Figure 6B**). Compared with Ductal 2-LL cells, Ductal 2-LH cells show increased MIF, NRG, and HGF pathways, while pathways such as BTLA, CD40, and LIGHT are decreased.

The neuregulin (NRG) signaling pathway exhibited marked heterogeneity. It showed minimal expression in malignant cells with low lactate metabolism but significantly higher expression in malignant bile duct cells with high lactate metabolism (ductal 2 LL). Given this variation, we focused on the NRG pathway for further analysis, examining communication dynamics among malignant cells with different metabolic states and interactions involving hyperlactate metabolic malignant cells (**Figure 6C**). We found that malignant cells with high lactate metabolism act as central mediators in the NRG pathway, closely communicating with Schwann cells and Ductal 2-LL, contributing to intercellular communication within the PDAC microenvironment (**Figures 6D, E**). These results highlight the potential importance of NRG signaling in PDAC metabolic reprogramming and progression, suggesting a metabolic-neural-immune tripartite interaction network and providing new evidence for key nodes that link metabolic reprogramming with neural invasion during PDAC development.

### 3.8 TF analysis in PDAC

**Figure 7:**
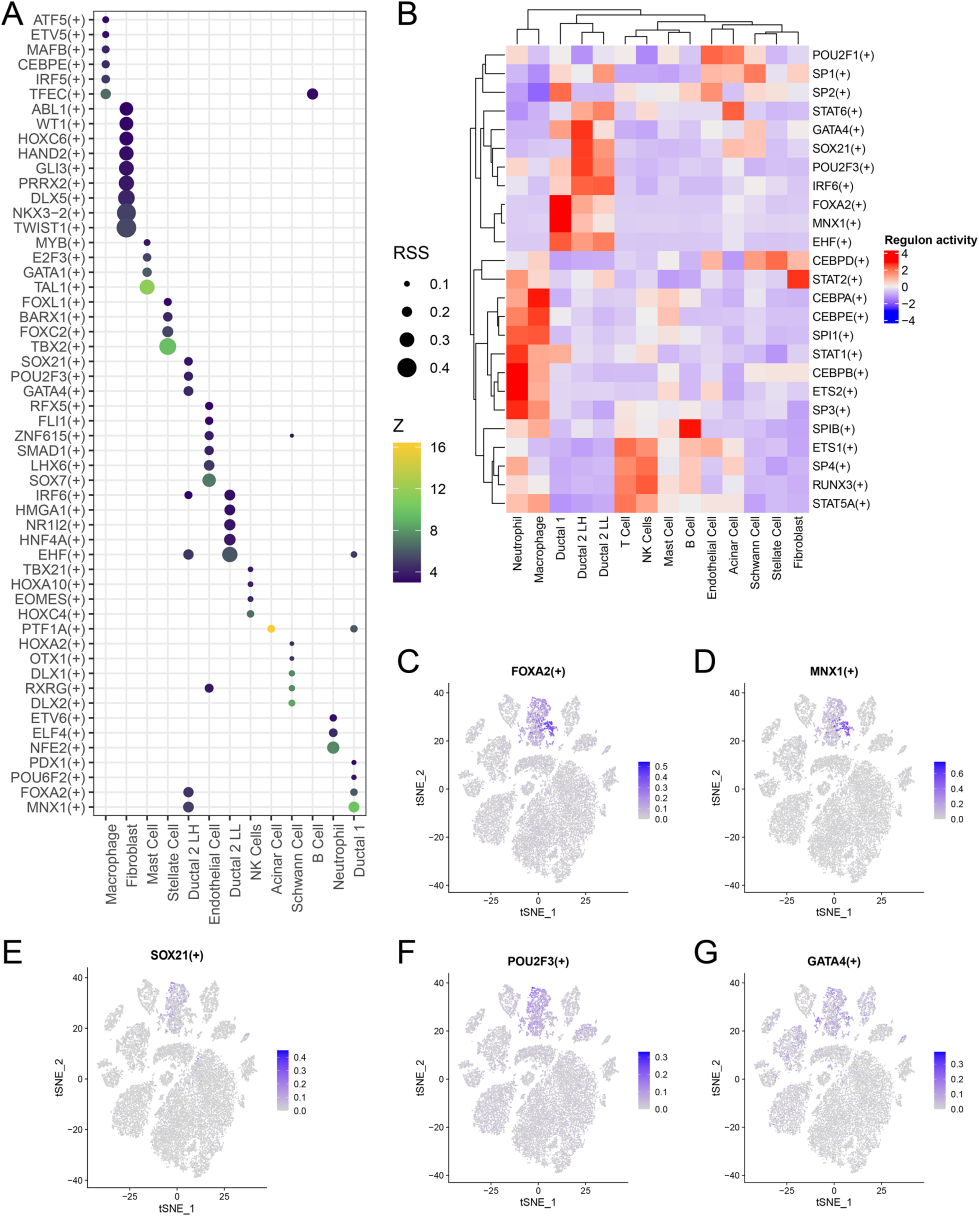
TF analysis in PDAC. **(A)** Bubble plot showing TFs specific to each cell type. **(B)** Heatmap illustrating expression patterns of cell type–specific TFs. **(C–G)** Representative expression patterns of FOXA2(+), MNX1(+), GATA4(+), POU2F3(+), and SOX21(+) in patients with PDAC. TF, transcription factor; PDAC, pancreatic ductal adenocarcinoma.

We employed SCENIC analysis to identify key TF modules and examine differences in TF expression between malignant cholangiocytes with distinct lactate-metabolic activity. Malignant bile duct cells with hyperlactate metabolism were predominantly associated with the following TF modules: FOXA2 (+), MNX1 (+), EHF (+), IRF6 (+), GATA4 (+), POU2F3 (+), and SOX21 (+) (**Figures 7A, B**). Conversely, bile duct cells with hypolactate metabolism were linked to the following TF modules: TBX21 (+), HOXA10 (+), EOMES (+), and HOXC (+) (**Figures 7A, B**). Additionally, FOXA2 (+), MNX1 (+), GATA4 (+), POU2F3 (+), and SOX21 (+) were enriched in malignant bile duct cells with hyperlactate metabolism (**Figures 7C–G**).

### 3.9 Drug sensitivity analysis

We also assessed the predictive value of the risk score for chemosensitivity in patients with PDAC. The clinical efficacy of several drugs, including dasatinib (1079), BI-2536 (1086), AZD8055 (1059), sepantronium bromide (1941), AZ6102 (2109), and bortezomib (1191), was evaluated for PDAC treatment potential. Patients with low-risk scores showed higher sensitivity to dasatinib (1079) (**Supplementary Figure 5A**), BI-2536 (1086) (**Supplementary Figure 5B**), and sepantronium bromide (1941) (**Supplementary Figure 5D**). In contrast, those with high risk were more sensitive to AZD8055 (1059) (**Supplementary Figure 5E**), AZ6102 (2109) (**Supplementary Figure 5C**), and bortezomib (1191) (**Supplementary Figure 5F**), a proteasome inhibitor with clinical efficacy in multiple myeloma treatment, as evidenced by improved progression-free survival and OS rates.

Additionally, LYZ expression correlated positively with sensitivity to dasatinib (1079) (R = 0.62, *p* <.001), BI-2536 (1086) (R = 0.55, *p* <.001), and sepantronium bromide (1941) (R = 0.37, *p* <.001) (**Supplementary Figures 5G–I**). Similarly, PIGR expression was positively correlated with sepantronium bromide (1941) (R = 0.58, *p* <.001) and BI-2536 (1086) (R = 0.66, *p* <.001) but negatively correlated with AZ6102 (2109) (R = 0.25, *p* = .01) (**Supplementary Figures 5J–L**).

### 3.10 Association of immune checkpoints and immune escape with biomarker genes

**Figure 8:**
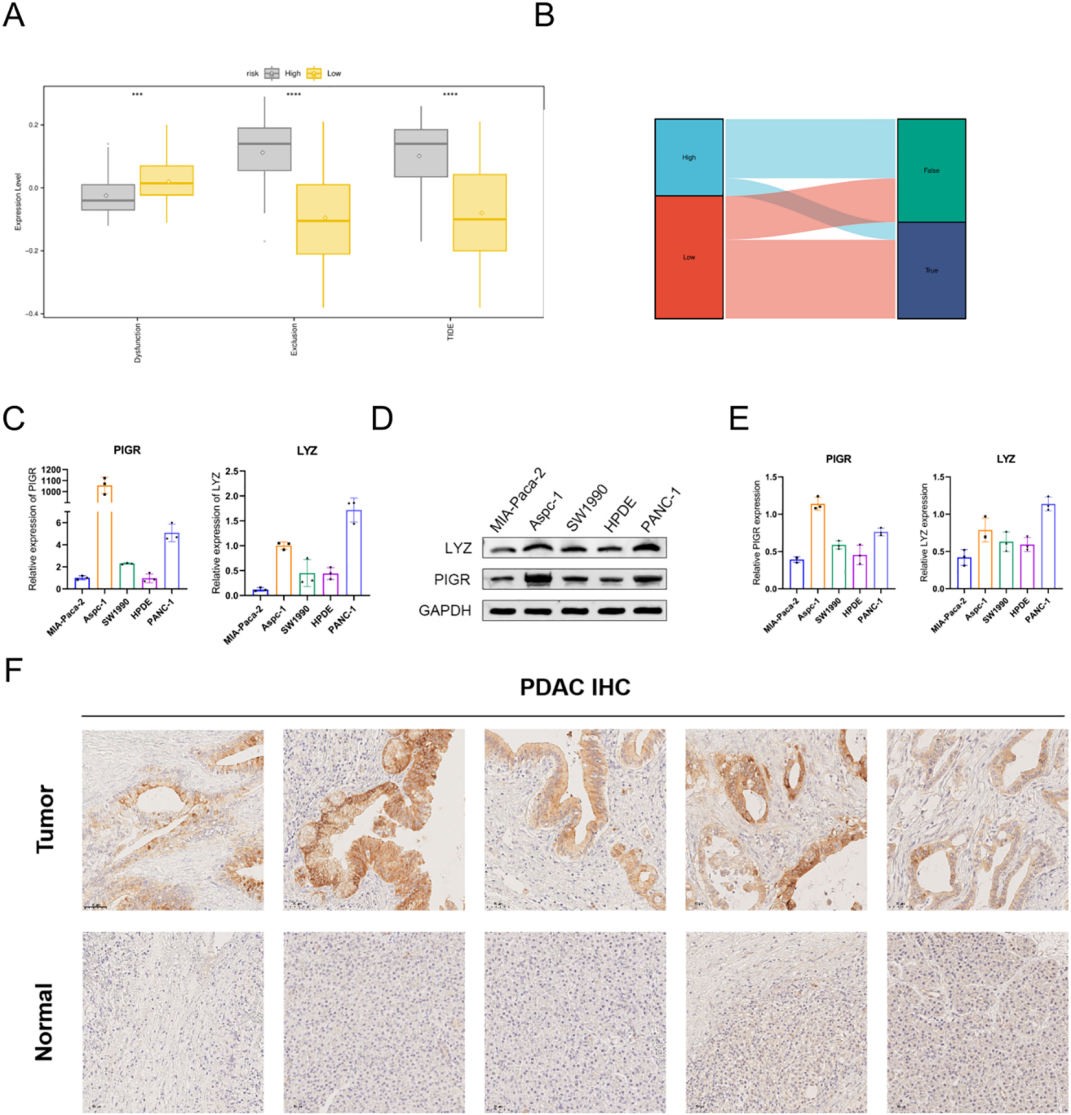
Immunocheckpoint, TIDE, and experimental validation analyses. **(A)** Bar chart showing differential TIDE scores between risk groups. **(B)** Sankey plot illustrating the relationship between risk score and immune response. **(C)** mRNA expression levels of *PIGR* and *LYZ* in ASPC-1, HPDE, MIA-PaCa-2, PANC-1, and SW1990 cell lines. **(D, E)** Protein expression of PIGR and LYZ in the same cell lines determined via western blot. **(F)** IHC showing high PIGR expression in PDAC tissues and low expression in adjacent normal tissues. Scale bars = 50 μm. TIDE, tumor immune dysfunction and exclusion; PDAC, pancreatic ductal adenocarcinoma; IHC, immunohistochemistry.

We analyzed the relationships between prognostic genes and immune checkpoints by comparing immune checkpoint expression levels in high- and low-expression groups for prognostic genes. BTLA, CD96, HHLA2, ILRB4, and TIGIT were significantly upregulated in the LYZ high-expression group (**Supplementary Figure 6A**). Similarly, HHLA2 and ILRB4 were significantly upregulated in the PIGR high-expression group (**Supplementary Figure 6B**). Tumor immune microenvironment heterogeneity critically influences tumor progression and patient prognosis, as evidenced in prior studies. To assess immune status between high- and low-risk groups, we compared TIDE and risk scores (**Figure 8A**). Results revealed higher TIDE and immune rejection scores in the high-risk group, suggesting an increased likelihood of immune escape (**Figure 8B**).

### 3.11 Pan-cancer analysis of prognostic genes

We examined PIGR mRNA expression across multiple cancer types compared with paired normal tissues. Consistent with previous studies on differential gene expression in sarcoma and endometrial carcinoma, PIGR expression was significantly elevated in these cancers, as well as in rectal adenocarcinoma, lung adenocarcinoma (LUAD), lung squamous cell carcinoma (LUSC), squamous cell carcinoma of the head and neck (HNSC), renal chromophobe carcinoma, renal clear cell carcinoma (KIRC), renal papillary cell carcinoma (KIRP), breast cancer (BRCA), and colon cancer (COAD) relative to normal tissues (**Supplementary Figure 7A**).

Conversely, LYZ expression was significantly elevated in BRCA, cholangiocarcinoma, COAD, glioblastoma, HNSC, KIRC, KIRP, and hepatocellular carcinoma but notably reduced in LUAD and LUSC (**Supplementary Figure 8A**). Furthermore, LYZ and PIGR expression levels were positively correlated with immune cell scores in most analyzed cancer types (**Supplementary Figures 7B**, **8B**).

### 3.12 Cell and tissue verification

We used five different cell lines to verify the expression of LYZ and PIGR, among which ASPC-1, MIA-PACA2, and PANC-1 are PDAC cell lines, while HPDE and SW1990 are control cell lines. RT-PCR analysis showed that PIGR mRNA expression was significantly higher in ASPC-1 cells compared with the other four cell lines, with highly significant pairwise differences detected (*p* <.001). LYZ mRNA expression was also significantly higher in ASPC-1 and PANC-1 cells compared with the other three cell types (*p* = .01), consistent with bioinformatics findings (**Figure 8C**).

Western blot analysis confirmed that PIGR expression was significantly elevated in ASPC-1 cells relative to the other cell types (*p* = .002). Similarly, LYZ expression was significantly higher in PANC-1 cells compared with the other cell types (*p* = .01). The concordance between experimental and computational results further validated the findings (**Figures 8D, E**). Additionally, immunohistochemical staining of paraffin-embedded PDAC tissue sections revealed minimal differences in LYZ expression between cancerous and adjacent normal tissues; however, PIGR expression was notably higher in cancerous tissues (**Figure 8F**).

## 4 Discussion

PDAC is a highly aggressive cancer, typically diagnosed at advanced stages and associated with poor prognosis, contributing substantially to global cancer mortality (21, 22). The complexity of its TME and pronounced genetic heterogeneity hinder effective therapy development (23). Advances in scRNA-seq have provided critical insights into PDAC’s cellular diversity and molecular mechanisms (24). Understanding metabolic alterations, particularly those involving lactate metabolism, is crucial for developing targeted treatments that improve prognosis (25). This study investigated PDAC cellular heterogeneity, emphasizing lactate metabolism, and identified potential biomarkers for prognosis and therapy.

We integrated single-cell transcriptomics, bulk RNA-seq, spatial transcriptomics, and experimental validation to analyze the PDAC TME. Accordingly, we identified two lactate-associated cell subtypes with distinct roles in disease progression and therapeutic response. Using advanced computational methods, we mapped dynamic interactions between these subtypes, suggesting novel TME-focused therapeutic strategies to improve prognosis.

In total, we identified 50, 795 cells divided into 26 clusters representing 13 major cell types, illustrating the TME’s complexity. InferCNV analysis distinguished malignant from nonmalignant cells, revealing 521 malignant cells with elevated lactate metabolism consistent with the Warburg effect (26, 27). Two lactate metabolism–related subtypes were defined: Cluster 1, characterized by NUPR1, TPM3, SERPINE1, and SLC2A1, expressed in both tumor and normal cells; and Cluster 2, characterized by B2M, CD24, LGALS3, and TM4SF1, mainly expressed in tumor and epithelial cells. Cluster 1 correlated with significantly better survival compared to Cluster 2, underscoring lactate metabolism’s role in PDAC progression.

Consensus clustering analysis revealed a differentiation continuum between subtypes, driven by metabolic activity and signaling pathways. Cluster 2, with higher lactate metabolism, exhibited greater tumor aggressiveness and a more inflammatory microenvironment conducive to invasion and metastasis. Patients in Cluster 2, with high lactate metabolism, had worse prognosis, reinforcing metabolic reprogramming as a driver of PDAC progression.

The PDAC immune microenvironment, strongly shaped by lactate metabolism, critically influences intercellular communication and immune regulation. The malignant ductal cell subset with high lactate metabolism identified in this study establishes paracrine communication with Schwann cells through the NRG signaling pathway, revealing a coupling mechanism between metabolic reprogramming and neural interactions in PDAC perineural invasion (PNI). The high-lactate phenotype reflects enhanced glycolysis driven by the Warburg effect, wherein lactate not only acidifies the microenvironment but also functions as a signaling molecule regulating gene expression through protein lactylation modifications (28). NRG1, secreted by cancer-associated fibroblasts (CAFs) and Schwann cells within the tumor microenvironment, activates the ErbB3 receptor on tumor cell surfaces and its downstream PI3K/AKT pathway, promoting proliferation and invasion (29). Upon tumor “hijacking, “ Schwann cells form tumor-activated Schwann cell tracks (TASTs), inducing tumor cell transition to basal-like subtypes through secretion of MDK and IL-1α, while establishing a bFGF/IL-33 positive feedback loop with tumor-associated macrophages (TAMs) (30, 31). This tripartite metabolic-neural-immune interaction network provides multidimensional support for PNI, suggesting that combinatorial targeting of lactate metabolism (e.g., LDHA/MCT inhibitors) and the NRG pathway (e.g., zenocutuzumab) may represent a therapeutic strategy for blocking perineural invasion.

SCENIC analysis identified key TFs (FOXA2, GATA4, and MNX1) as regulators of high lactate metabolism. Although FOXA2’s metabolic role in PDAC was previously described, we uniquely demonstrate its coregulation with lactate transporters (MCT1/4) and immune checkpoints (PD-L1) (32–34). Conversely, low-lactate-associated TFs (TBX21 and HOXA10) showed enrichment patterns similar to the “immune-hot” PDAC subtype described by Ma et al.; however, our study is the first to link these TFs to metabolic suppression (35–37).

PIGR emerged as a novel lactate-modulated target in PDAC. Although its immunosuppressive function was reported in colorectal cancer, our study provides the first comprehensive evidence of its importance in PDAC. We validated its marked overexpression in PDAC compared with normal tissues (*p* <.001), noting a difference greater than that observed in colorectal cancer, and confirmed its association with NF-κB activation, a pathway implicated in PDAC chemoresistance (38–40). Our findings suggest that PIGR is associated with pathways such as NF-κB activation and EMT, but functional studies are needed to determine causality.

For LYZ, beyond its known immune functions, pathway analysis revealed novel associations with KRAS signaling, supporting evidence of KRAS-driven metabolic reprogramming in PDAC (41). LYZ expression is enriched in gene sets associated with KRAS signaling and IL2–STAT5 pathways (42).

This study represents a fundamental advance in PDAC research: spatial transcriptomics, for the first time, revealed spatial colocalization between lactate metabolism–associated TFs and immune checkpoint molecules, a finding not achievable through bulk RNA-seq (43). The PIGR–NF-κB–EMT axis described in this study provides a mechanistic connection between the “fibro-inflammatory” PDAC subtype and metabolic heterogeneity (44).

Finally, lactate metabolism–related genes emerged as markers of malignant cell behavior, with high lactate–producing cells correlating with poorer clinical outcomes (45). Targeting lactate metabolism may offer a new therapeutic strategy by disrupting tumor-supportive metabolic adaptations. Our pharmacogenomic analysis showed that patients with low-risk scores are more sensitive to dasatinib and BI-2536, whereas high-risk patients responded better to AZD8055 and bortezomib. Expression levels of LYZ and PIGR were linked to drug sensitivity, highlighting their potential as biomarkers for PDAC treatment.

Despite these insights, this study has several limitations. These include potential bias from public datasets and the relatively small experimental validation cohort. Larger, more uniform patient cohorts with detailed clinical data are required to refine and confirm these findings. Moreover, machine learning models must be validated on independent cohorts to ensure prognostic accuracy (46, 47).

## 5 Conclusion

In summary, we identified two genes, PIGR and LYZ, closely associated with lactate metabolism in PDAC through integrated bioinformatics analysis. Validation confirmed significant PIGR overexpression in PDAC compared with adjacent normal tissues, establishing it as a key biomarker for lactate metabolism. These genes appear to regulate PDAC progression, making PIGR and LYZ promising therapeutic targets. Our findings provide a foundation for future clinical strategies targeting lactate metabolism and offer potential new directions for PDAC treatment.

## Data Availability

The datasets presented in this study can be found in online repositories. The names of the repository/repositories and accession number(s) can be found in the article/**Supplementary Material**.
