# Supplementary figures and images for: Spatial transcriptomics reveals the mechanistic role of lactate metabolism in the pancreatic ductal adenocarcinoma microenvironment
Source: Front Immunol. 2026 Feb 13;17:1743187. doi: 10.3389/fimmu.2026.1743187 (PMC12946077; doi:10.3389/fimmu.2026.1743187)

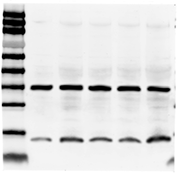

Supplement: Supplementary file 1 [file Image1.tif]

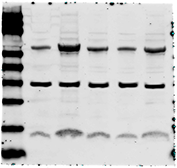

Supplement: Supplementary file 2 [file Image2.tif]

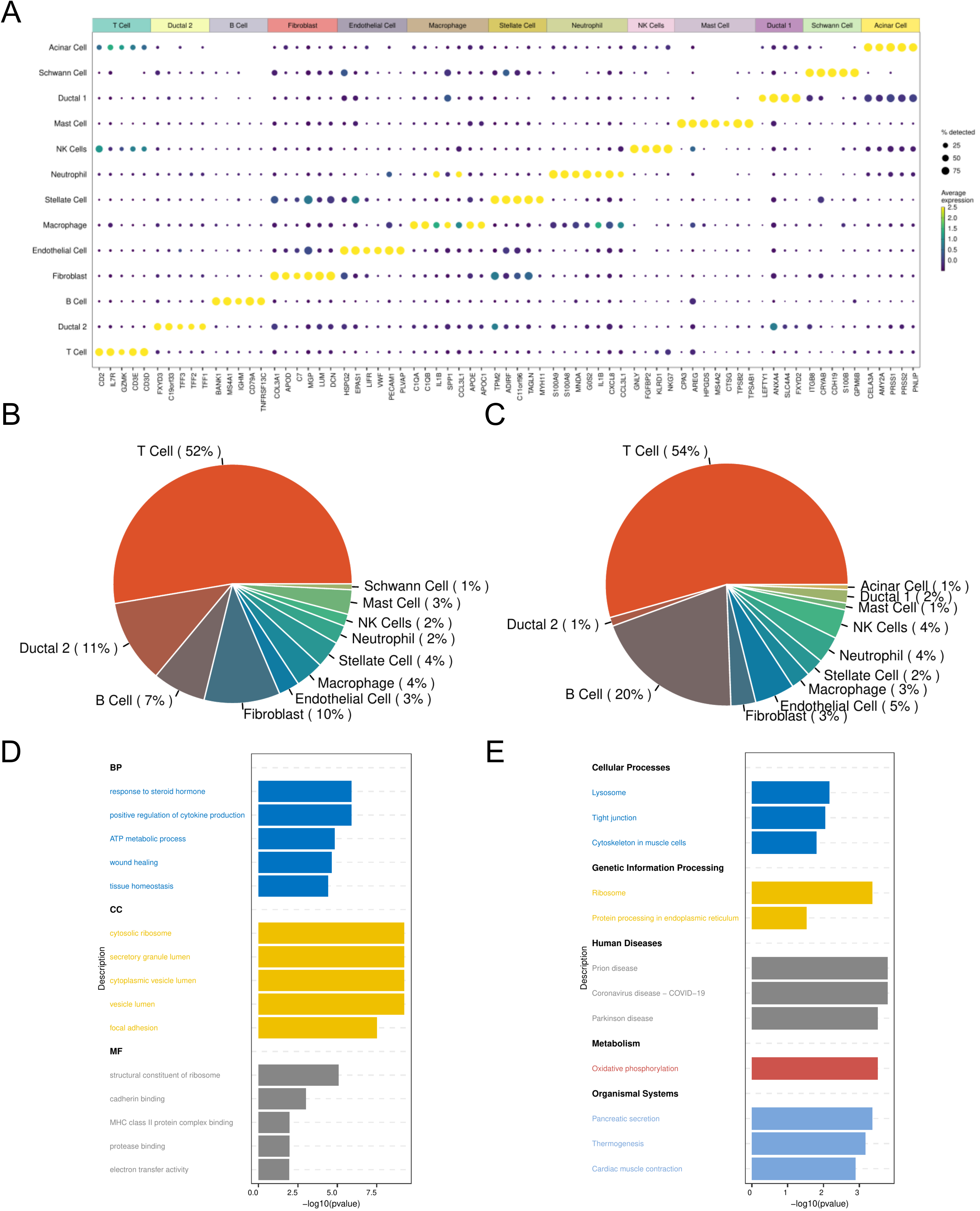

Supplement: Supplementary file 3 [file Image3.tiff]

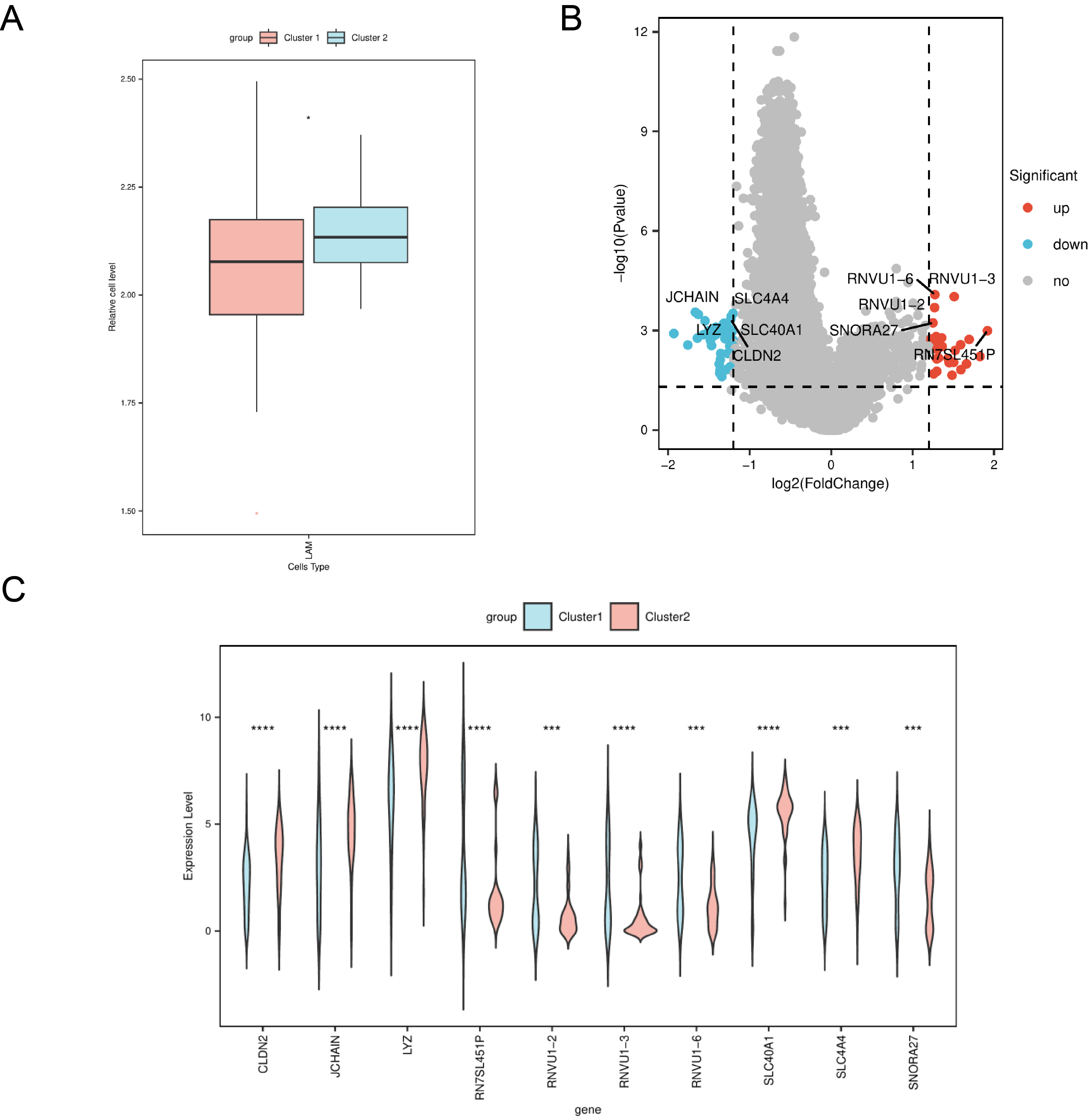

Supplement: Supplementary file 4 [file Image4.tif]

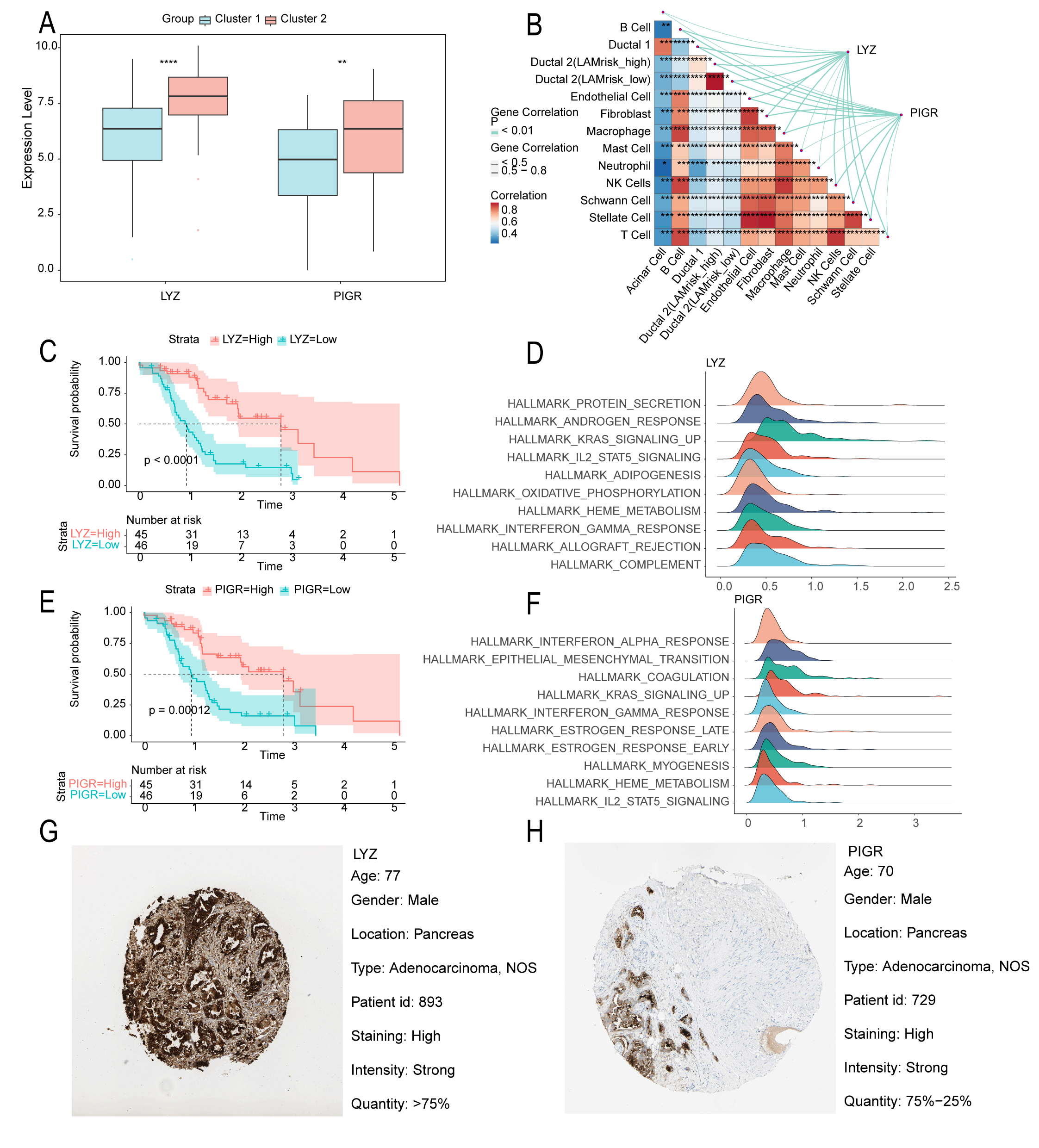

Supplement: Supplementary file 5 [file Image5.tif]

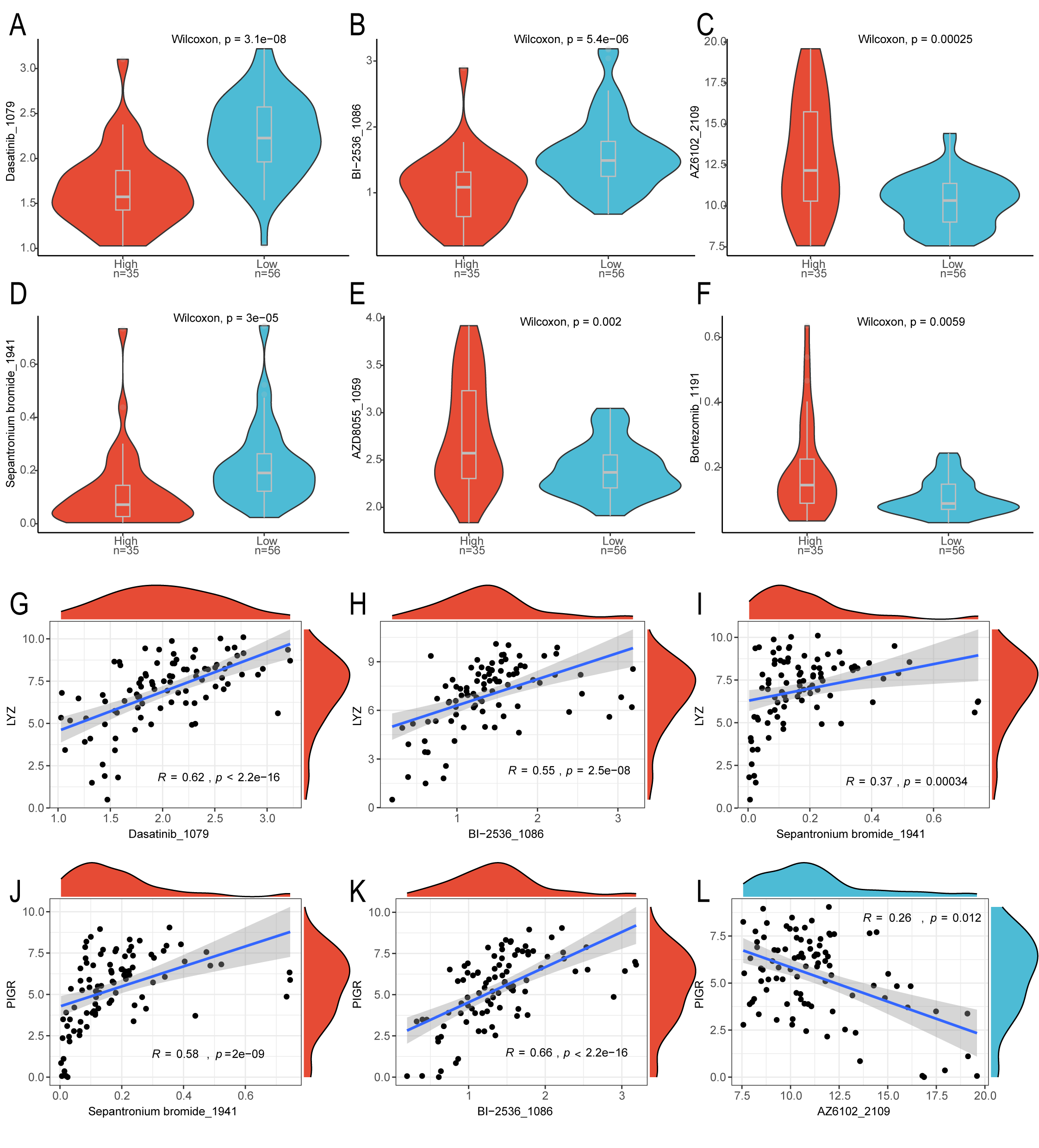

Supplement: Supplementary file 6 [file Image6.tif]

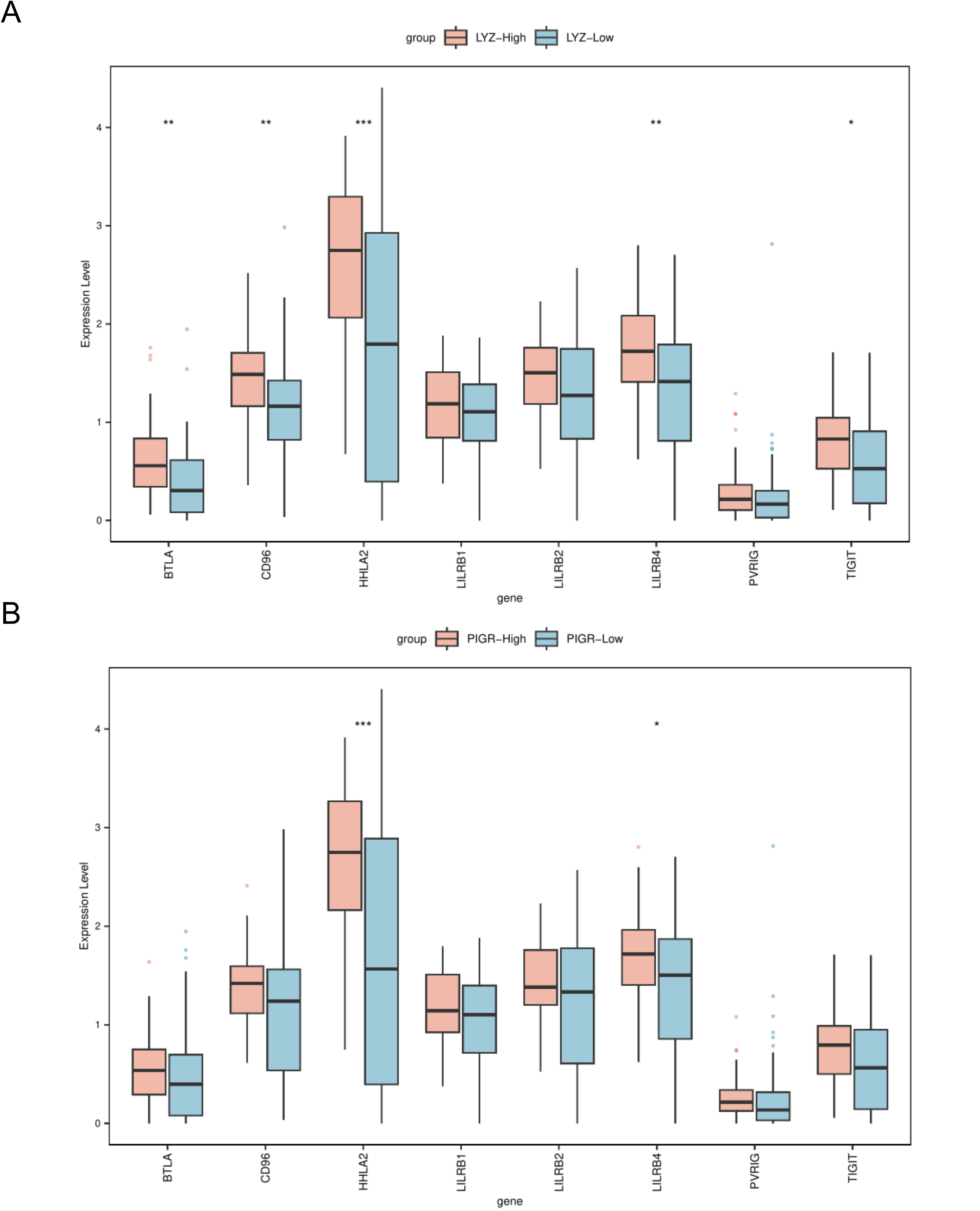

Supplement: Supplementary file 7 [file Image7.tiff]

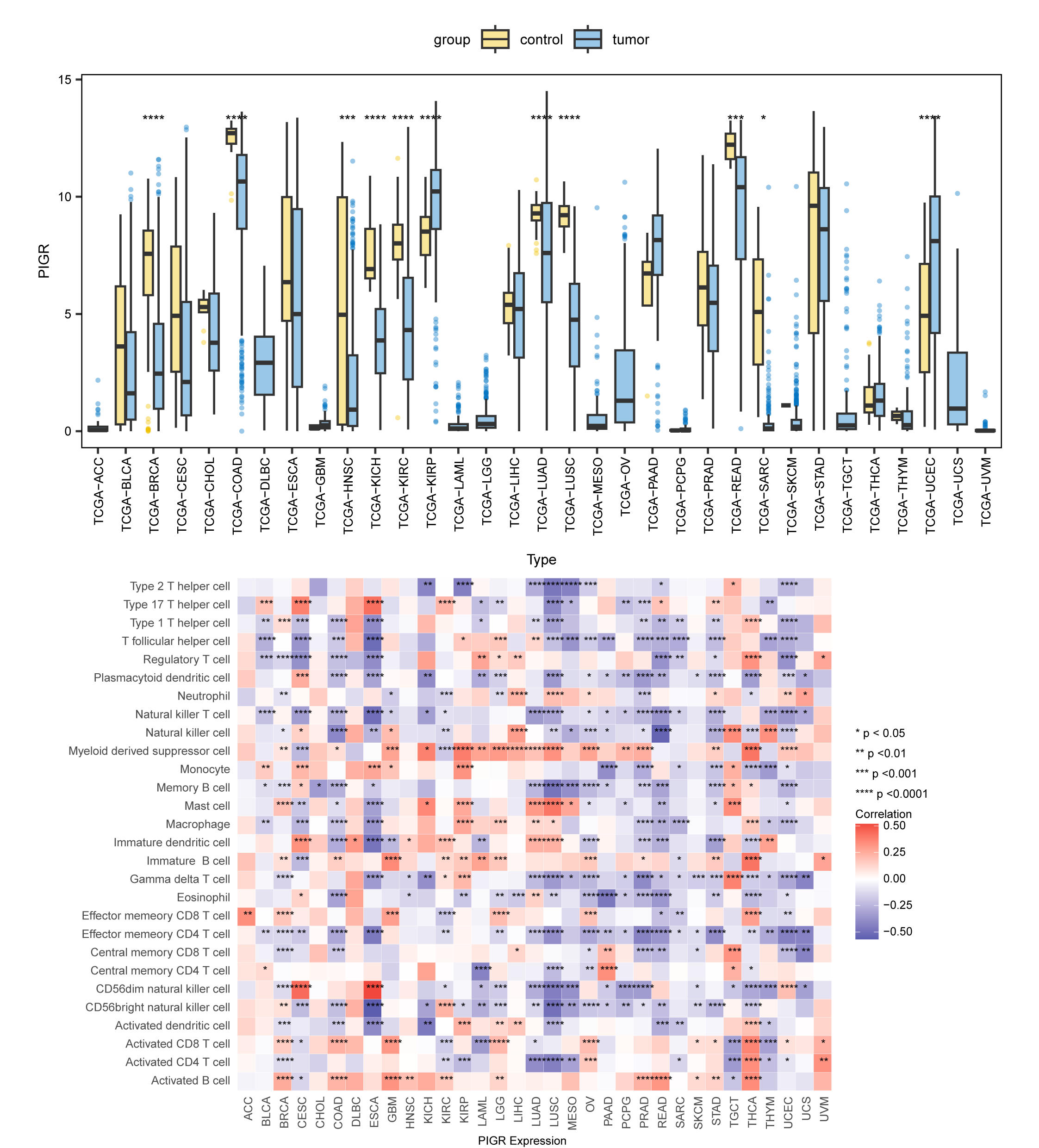

Supplement: Supplementary file 8 [file Image8.tif]

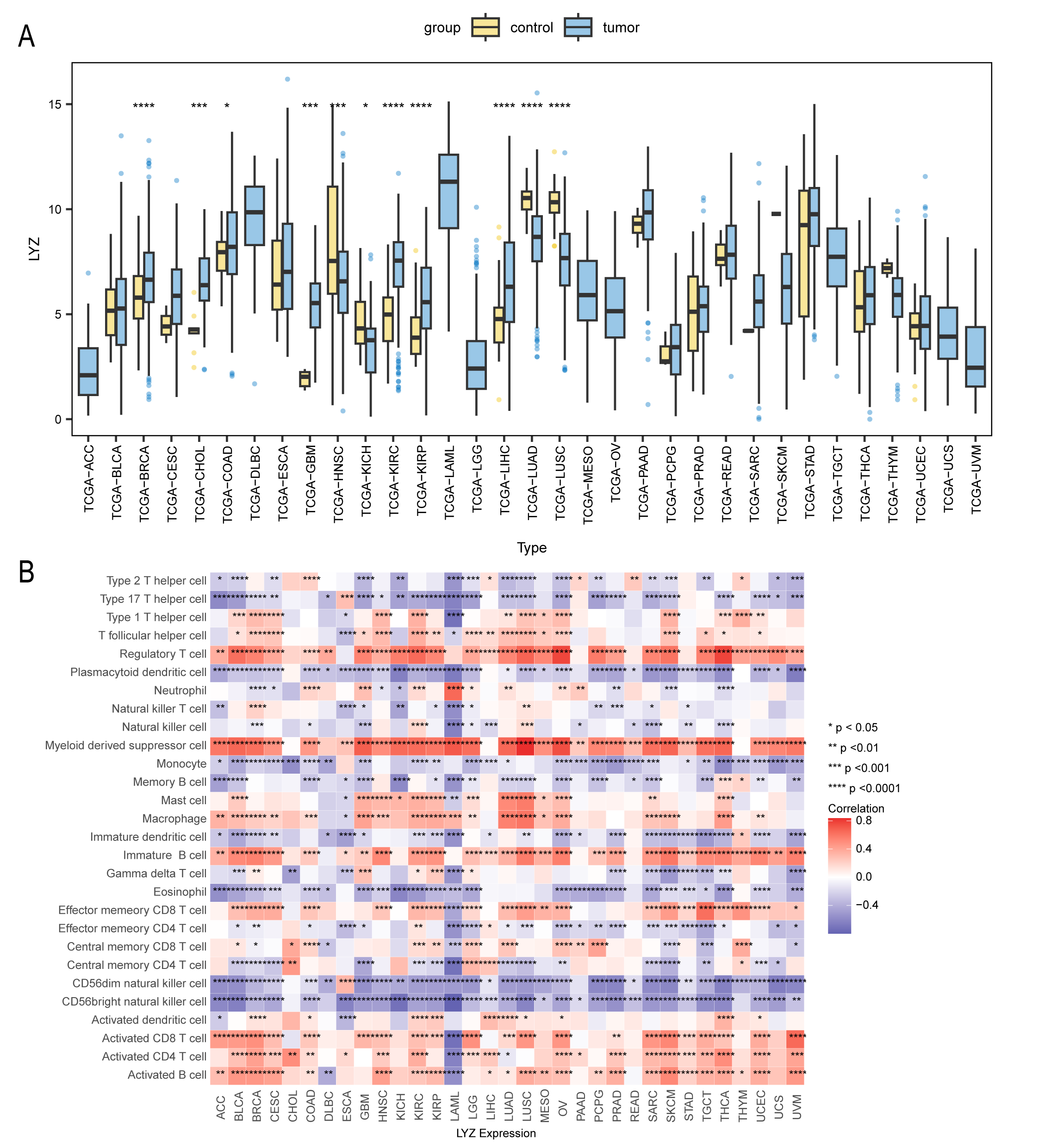

Supplement: Supplementary file 9 [file Image9.tif]

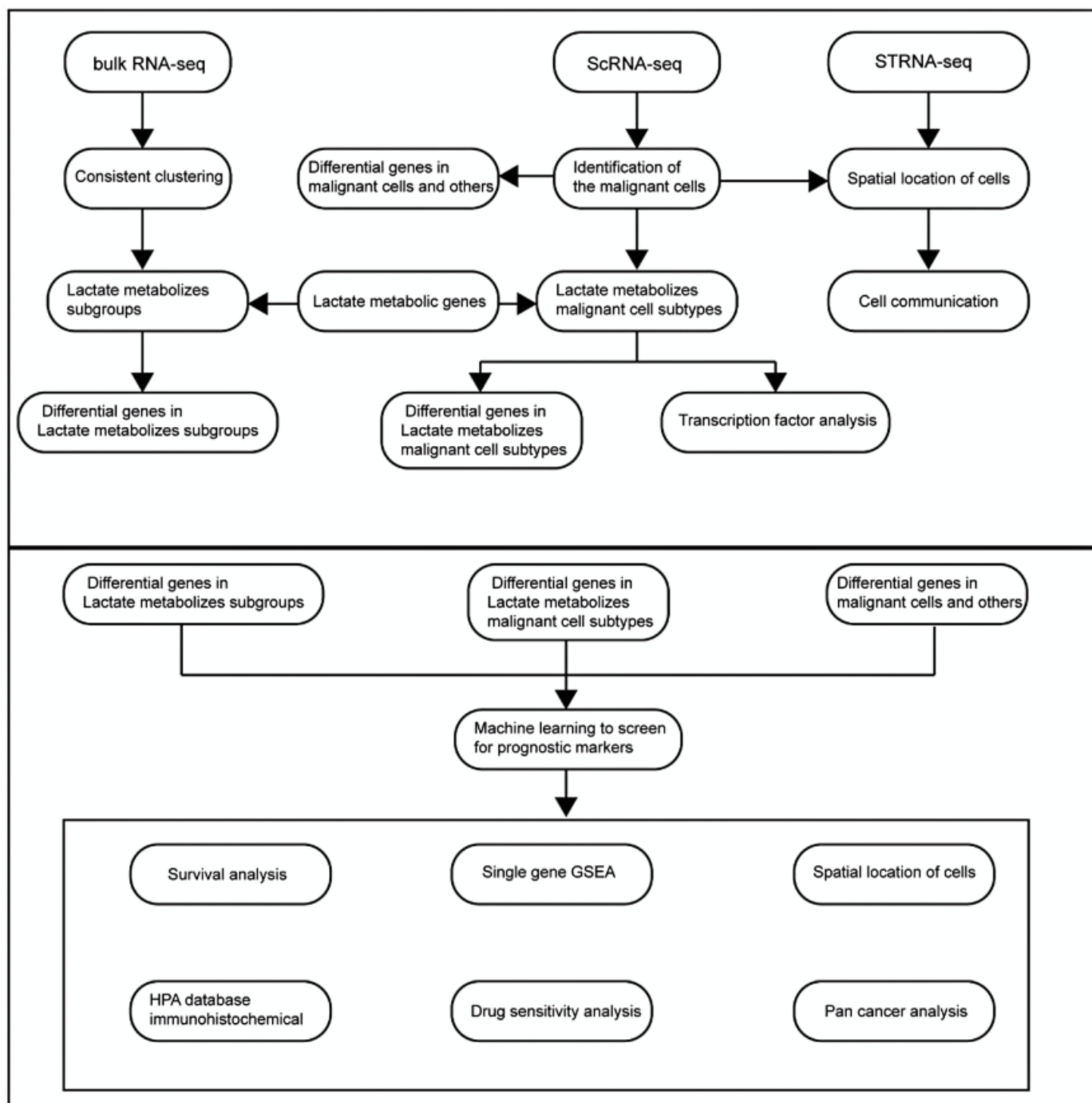

Supplementary Figure\_1 Flow-process diagram of this study

Supplement: Supplementary file 20 [file DataSheet1.pdf]
