# Supplementary material for: Spatial transcriptomics reveals the mechanistic role of lactate metabolism in the pancreatic ductal adenocarcinoma microenvironment
Source: Front Immunol. 2026 Feb 13;17:1743187. doi: 10.3389/fimmu.2026.1743187 (PMC12946077; doi:10.3389/fimmu.2026.1743187)
Supplement: Supplementary file 10 [file Table1.docx]

lactate metabolism-related gene

AARS2

ACAD9

ACAT1

ACAT2

ACTN3

ADAMTS13

AGK

AIFM1

ALDH6A1

ALDH7A1

ALDOB

AMPD1

AMPD2

AMPD3

ATAD3A

ATP5F1A

ATP5F1D

ATP5F1E

ATP5MK

ATPAF1

ATPAF2

BCKDHA

BCKDHB

BCS1L

BOLA3

C1QBP

CA5A

CAMKMT

CARS2

CDK5

CHCHD10

CLPB

COA6

COA8

COQ2

COQ4

COQ8A

COQ9

COX10

COX11

COX14

COX15

COX16

COX20

COX4I1

COX5A

COX6A2

COX6B1

COX8A

CRAT

CYC1

CYP27A1

DARS2

DBT

DGUOK

DLAT

DLD

DNAJC19

DNM1L

DTYMK

EARS2

ECHS1

ELAC2

ETHE1

FARS2

FASTKD2

FBP1

FBXL4

FDX2

FH

FOXRED1

G6PC1

GATB

GATC

GATD1

GATM

GFER

GFM1

GFM2

GLRX5

GOT2

GTPBP3

GYS2

HADH

HADHA

HADHB

HAGH

HIBCH

HIF1A

HLCS

HMGCL

HPDL

HS6ST2

HSD17B10

HSPD1

HTRA2

IBA57

ISCA1

ISCU

KARS1

LARS1

LARS2

LDHA

LDHAL6A

LDHAL6B

LDHB

LDHC

LDHD

LETM1

LIAS

LIG3

LIPT1

LIPT2

LONP1

LRPPRC

LYRM4

LYRM7

MARS1

MDH2

MECP2

MICOS13

MIPEP

MIR210

MLYCD

MPC1

MPV17

MRPL12

MRPL3

MRPL44

MRPS14

MRPS16

MRPS2

MRPS22

MRPS28

MRPS34

MRPS7

MRS2

MT-ATP6

MT-ATP8

MT-CO1

MT-CO2

MT-CO3

MT-CYB

MT-ND1

MT-ND2

MT-ND3

MT-ND4

MT-ND5

MT-ND6

MT-TC

MT-TE

MT-TF

MT-TH

MT-TI

MT-TK

MT-TL1

MT-TL2

MT-TN

MT-TP

MT-TQ

MT-TS1

MT-TS2

MT-TT

MT-TV

MT-TW

MTFMT

MTO1

MTRFR

NADK2

NARS2

NAXD

NAXE

NDUFA1

NDUFA10

NDUFA11

NDUFA12

NDUFA13

NDUFA4

NDUFA6

NDUFA8

NDUFA9

NDUFAF1

NDUFAF2

NDUFAF3

NDUFAF4

NDUFAF5

NDUFAF6

NDUFAF8

NDUFB10

NDUFB11

NDUFB3

NDUFB7

NDUFB8

NDUFB9

NDUFC2

NDUFS1

NDUFS2

NDUFS3

NDUFS4

NDUFS6

NDUFS7

NDUFS8

NDUFV1

NDUFV2

NFS1

NFU1

NGLY1

NSUN3

NUBPL

OCRL

OGDH

PARK7

PC

PCCA

PCCB

PCK1

PDHA1

PDHB

PDHX

PDP1

PDSS1

PDSS2

PER2

PET100

PET117

PFKFB2

PHKA2

PHKB

PHKG2

PITRM1

PLPBP

PMPCA

PMPCB

PNKD

PNPLA8

PNPO

PNPT1

POLG

POLG2

POLRMT

PPCS

PPM1B

PREPL

PRORP

PRSS12

PUS1

PYGL

QRSL1

RARS1

RARS2

RMND1

RNASEH1

RRM2B

RYR1

SARS2

SCO1

SCO2

SDHA

SDHB

SDHD

SERAC1

SFXN4

SLC25A10

SLC25A12

SLC25A13

SLC25A19

SLC25A21

SLC25A26

SLC25A3

SLC25A4

SLC25A42

SLC31A1

SLC37A4

SLC3A1

SLC52A1

SLC7A7

SOD1

SQOR

STAT2

SUCLA2

SUCLG1

SURF1

SYNJ1

TAFAZZIN

TANGO2

TARS2

TIGAR

TIMM22

TIMM50

TIMMDC1

TK2

TKFC

TMEM126B

TMEM70

TP53

TPK1

TRMT10C

TRMT5

TRMU

TRNT1

TSFM

TTC26

TUFM

TWNK

TXN2

TYMP

UPB1

UQCC2

UQCC3

UQCRB

UQCRC2

UQCRFS1

UQCRH

UQCRQ

USP18

VARS2

WARS2

YARS1

YARS2

YME1L1
