# Supplementary material for: Spatial transcriptomics reveals the mechanistic role of lactate metabolism in the pancreatic ductal adenocarcinoma microenvironment
Source: Front Immunol. 2026 Feb 13;17:1743187. doi: 10.3389/fimmu.2026.1743187 (PMC12946077; doi:10.3389/fimmu.2026.1743187)
Supplement: Supplementary file 11 [file Table2.docx]

"celltype" "marker"

"Schwann Cell" "S100B"

"Schwann Cell" "CDH19"

"Schwann Cell" "GPM6B"

"Schwann Cell" "ITGB8"

"Schwann Cell" "CRYAB"

"Mast Cell" "TPSB2"

"Mast Cell" "CPA3"

"Mast Cell" "TPSAB1"

"Mast Cell" "MS4A2"

"Mast Cell" "HPGDS"

"Mast Cell" "CTSG"

"Mast Cell" "AREG"

"Endothelial Cell" "PLVAP"

"Endothelial Cell" "VWF"

"Endothelial Cell" "PECAM1"

"Endothelial Cell" "HSPG2"

"Endothelial Cell" "EABP5"

"Endothelial Cell" "EPAS1"

"Endothelial Cell" "LIFR"

"Stellate Cell" "C11orf96"

"Stellate Cell" "ADIRF"

"Stellate Cell" "MYH11"

"Stellate Cell" "TAGLN"

"Stellate Cell" "TPM2"

"Neutrophil" "CXCL8"

"Neutrophil" "G0S2"

"Neutrophil" "CCL3L1"

"Neutrophil" "IL1B"

"Neutrophil" "S100A9"

"Neutrophil" "S100A8"

"Neutrophil" "MNDA"

"Neutrophil" "FCGR38"

"B Cell" "MS4A1"

"B Cell" "CD79A"

"B Cell" "IGHM"

"B Cell" "TNFRSF13C"

"B Cell" "BANK1"

"Macrophage" "APOE"

"Macrophage" "APOC1"

"Macrophage" "SPP1"

"Macrophage" "C1QA"

"Macrophage" "C1QB"

"Macrophage" "CCL3L1"

"Macrophage" "IL1B"

"Ductal 1" "FXYD2"

"Ductal 1" "SLC4A4"

"Ductal 1" "ANXA4"

"Ductal 1" "LEFTY1"

"Ductal 2" "FXYD3"

"Ductal 2" "TFF1"

"Ductal 2" "TFF3"

"Ductal 2" "TFF2"

"Ductal 2" "C19orf33"

"T Cell" "IL7R"

"T Cell" "CD3D"

"T Cell" "CD2"

"T Cell" "GZMK"

"T Cell" "CD3E"

"Fibroblast" "DCN"

"Fibroblast" "LUM"

"Fibroblast" "COL3A1"

"Fibroblast" "COL3A2"

"Fibroblast" "APOD"

"Fibroblast" "MGP"

"Fibroblast" "C7"

"Acinar Cell" "PRSS1"

"Acinar Cell" "PRSS2"

"Acinar Cell" "AMY2A"

"Acinar Cell" "CELA3A"

"Acinar Cell" "PNLIP"

"NK Cells" "GNLY"

"NK Cells" "NKG7"

"NK Cells" "FGFBP2"

"NK Cells" "KLRD1"
